# Phenotypic effects of mutations observed in the neuraminidase of human origin H5N1 influenza A viruses

**DOI:** 10.1371/journal.ppat.1011135

**Published:** 2023-02-06

**Authors:** David Scheibner, Ahmed H. Salaheldin, Ola Bagato, Luca M. Zaeck, Ahmed Mostafa, Ulrike Blohm, Christin Müller, Ahmed F. Eweas, Kati Franzke, Axel Karger, Alexander Schäfer, Marcel Gischke, Donata Hoffmann, Solène Lerolle, Xuguang Li, Hatem S. Abd El-Hamid, Jutta Veits, Angele Breithaupt, Geert-Jan Boons, Mikhail Matrosovich, Stefan Finke, Stephan Pleschka, Thomas C. Mettenleiter, Robert P. de Vries, Elsayed M. Abdelwhab

**Affiliations:** 1 Institute of Molecular Virology and Cell Biology, Friedrich-Loeffler-Institut, Federal Research Institute for Animal Health, Greifswald-Insel Riems, Germany; 2 Department of Poultry Diseases, Faculty of Veterinary Medicine, Alexandria University, El-Beheira, Egypt; 3 Center of Scientific Excellence for Influenza Viruses, National Research Centre (NRC), Water Pollution Research Department, Dokki, Giza, Egypt; 4 Institute of Immunology, Friedrich-Loeffler-Institut, Federal Research Institute for Animal Health, Greifswald-Insel Riems, Germany; 5 Institute of Medical Virology, Justus Liebig University Giessen, Giessen, Germany; 6 Department of Medicinal Chemistry, National Research Center, Dokki, Giza, Egypt; Department of Science, University of Technology and Applied Sciences-Rustaq, Rustaq, Sultanate of Oman; 7 Institute of Infectology, Friedrich-Loeffler-Institut, Federal Research Institute for Animal Health, Greifswald-Insel Riems, Germany; 8 Institute of Diagnostic Virology, Friedrich-Loeffler-Institut, Federal Research Institute for Animal Health, Greifswald-Insel Riems, Germany; 9 Centre for Biologics Evaluation, Biologics and Genetic Therapies Directorate, HPFB, Health Canada, Ottawa, ON, Canada; Department of Biochemistry, Microbiology and Immunology and Emerging Pathogens Research Centre, University of Ottawa, Ottawa, Ontario, Canada; 10 Department of Poultry Diseases, Faculty of Veterinary Medicine, Damanhur University, Al-Buheira, Egypt; 11 Department of Experimental Animal Facilities and Biorisk Management, Friedrich-Loeffler-Institut, Federal Research Institute for Animal Health, Greifswald-Insel Riems, Germany; 12 Department of Chemical Biology & Drug Discovery, Utrecht Institute for Pharmaceutical Science, the Netherlands; 13 Institute of Virology, Philipps University, Marburg, Germany; 14 German Center for Infection Research (DZIF) partner site Giessen-Marburg-Langen, Germany; 15 Friedrich-Loeffler-Institut, Federal Research Institute for Animal Health, Greifswald-Insel Riems, Germany; Emory University School of Medicine, UNITED STATES

## Abstract

Global spread and regional endemicity of H5Nx Goose/Guangdong avian influenza viruses (AIV) pose a continuous threat for poultry production and zoonotic, potentially pre-pandemic, transmission to humans. Little is known about the role of mutations in the viral neuraminidase (NA) that accompanied bird-to-human transmission to support AIV infection of mammals. Here, after detailed analysis of the NA sequence of human H5N1 viruses, we studied the role of A46D, L204M, S319F and S430G mutations in virus fitness *in vitro* and *in vivo*. Although H5N1 AIV carrying avian- or human-like NAs had similar replication efficiency in avian cells, human-like NA enhanced virus replication in human airway epithelia. The L204M substitution consistently reduced NA activity of H5N1 and nine other influenza viruses carrying NA of groups 1 and 2, indicating a universal effect. Compared to the avian ancestor, human-like H5N1 virus has less NA incorporated in the virion, reduced levels of viral NA RNA replication and NA expression. We also demonstrate increased accumulation of NA at the plasma membrane, reduced virus release and enhanced cell-to-cell spread. Furthermore, NA mutations increased virus binding to human-type receptors. While not affecting high virulence of H5N1 in chickens, the studied NA mutations modulated virulence and replication of H5N1 AIV in mice and to a lesser extent in ferrets. Together, mutations in the NA of human H5N1 viruses play different roles in infection of mammals without affecting virulence or transmission in chickens. These results are important to understand the genetic determinants for replication of AIV in mammals and should assist in the prediction of AIV with zoonotic potential.

## Author summary

Avian influenza viruses (AIV) including viruses of the H5Nx Goose/Guangdong-lineage caused hundreds of human infections and fatalities worldwide and pose a continuous zoonotic and pandemic threat. It is important to understand the role of mutations that support AIV-infection in mammals after bird-to-human transmission. Here, we describe the prevalence and the role of mutations in the viral neuraminidase (NA), which is mainly responsible for virus release from infected cells, preferentially selected in human H5N1 viruses. Compared to their avian ancestors, human-like H5N1 had less NA in virus particles and exhibited lower NA activity conferred mainly by the L204M substitution. Furthermore, NA mutations increased the binding of H5N1 to human-type receptors, acted synergistically to confer high virus replication in avian and human cells and/or modulated virulence and replication in mice, but not in chickens. This study is important to understand the genetic and biological properties of human AIV and will help to predict zoonotic potential AIV.

## Introduction

Influenza A viruses (IAV) possess a negative-sense RNA genome composed of eight segments, which encode at least 11 viral proteins. IAV are classified according to the variation of the surface glycoproteins hemagglutinin (HA) and neuraminidase (NA), which occur in 18 (for HA) and 11 (for NA) subtypes. IAV infect a wide range of species including birds and humans. Human influenza viruses belong to H1-H3 subtypes, while aquatic birds are the reservoir for H1-H16 avian influenza viruses (AIV) [1]. Although AIV generally do not replicate efficiently in humans, some strains are able to cross the species barrier and infect humans inducing infections which range from self-limiting flu-like illness to death [1]. Importantly, most of the pandemic human influenza viruses carried gene segments encoding HA, NA and/or polymerase basic-1 (PB1) from AIV [2,3]. Animal-to-human transmission of AIV has been accompanied by genetic alterations, which enabled efficient virus replication in human cells. While mutations linked to human-adaptation of AIV in PB2, PB1 and HA are well characterized [4,5], surprisingly little is known about the role of NA in human infections with AIV. NA is a tetrameric mushroom-like glycoprotein complex with each monomer composed of N-terminal, transmembrane, stalk and head domains [6]. The head domain contains the sialidase activity, which cleaves sialic acid (SA) to release progeny virions or digests mucin in the respiratory tract to enable systemic virus spread. The NA enzymatic pocket is stabilized by highly conserved catalytic and framework residues [6]. Therefore, neuraminidase inhibitors (NAI) are used successfully to control influenza viruses in humans [7]. Mutations in NA are thought to evolve to maintain a meticulous functional balance with the receptor-binding activity of the HA [8] or are selected for by external pressure (e.g. antivirals or vaccination) [9,10].

Since 1997, highly pathogenic (HP) AIV Goose/Guangdong (GsGd) H5Nx viruses continue to cause high losses in poultry and pose a serious pandemic risk [11]. The NA of GsGd H5N1 viruses usually possesses a NA-stalk deletion, a major determinant for adaptation of AIV in poultry [12], and the HA has evolved into 10 phylogenetic clades (clade 0 to 9) and tens of subclades. Clade 2 viruses, including the recent panzootic clade 2.3.4.4, are still endemic in Asia and Egypt [13]. Since 2006, Egypt has reported the highest number of GsGd-H5N1-infections in humans worldwide with ~42% (359/861) of infections and ~26% (120/455) of global fatal cases [14]. Compared to avian-origin H5N1 viruses, the Egyptian human-like (HL) H5N1 clade 2.2.1.2 viruses possessed HA mutations which enhanced virus affinity to the human-type 2,6-SA receptors but retained its interaction with avian-type 2,3-SA receptors [15], and mutations in the viral polymerase complex (PB2, PB1, PA, NP) which increased viral replication in human cells [16]. In 2014–2015, a novel group in clade 2.2.1.2 spread in poultry and transmitted to at least 165 humans [17,18]. Compared to the parental clade 2.2.1 in 2006 (designated avian-like, AL), the NA gene of HL-H5N1 in 2008 (HL-08; referring to human-origin H5N1 viruses in 2008) and 2015–2016 (HL-16; referring to human-origin H5N1 viruses in 2015/2016) viruses possessed 4 and 16 NA missense mutations, respectively, with unknown biological relevance [17].

Here, we analyzed all NA gene sequences of H5N1 viruses of human- and poultry-origin from Eurasia and Africa. We generated several recombinant viruses with mutations in the NA and studied their effect *in vitro* and *in vivo* in chickens, mice and ferrets. We found that NA mutations in human H5N1 play diverse roles in virus replication in mammals without affecting virus fitness in birds.

## Results

### Unique mutations co-evolved in the NA of human H5N1 viruses

**Fig 1:**
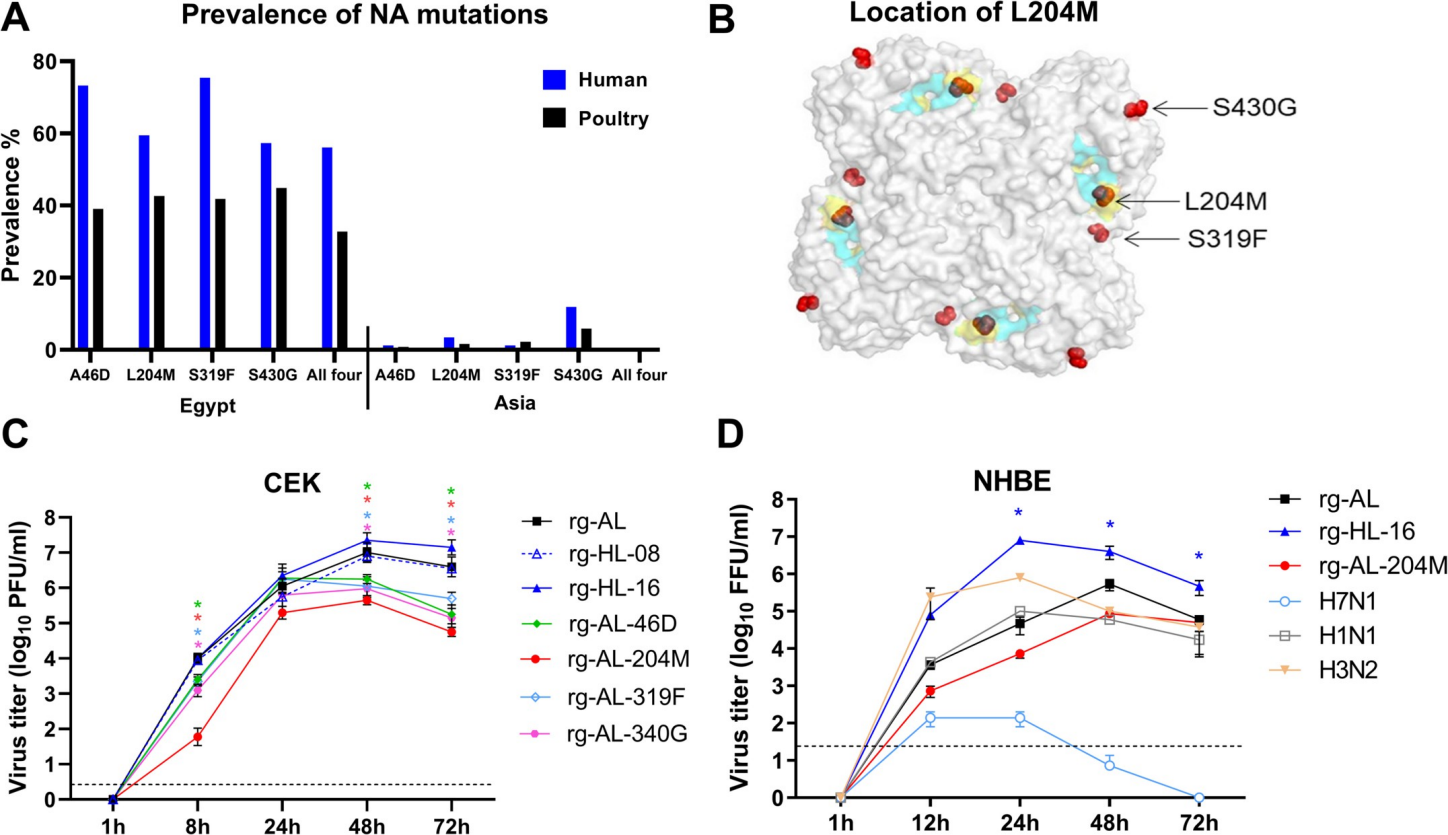
Prevalence of NA mutations in H5N1 of human and poultry-origin, structural modelling and replication in primary avian and human cell culture. Prevalence of N1-NA mutations in human (blue) and poultry (black) origin sequences of H5N1 in Egypt and Asia in GenBank and GISAID at 31.12.2016 were retrieved and analyzed using Geneious (A). Predicted location of NA mutations (depicted in red) on the NA tetramer generated by SWISS Model using the amino acid sequence of A/chicken/Egypt/06207/2006 (accession number ACR56180); one of the earliest viruses introduced into Egypt in February 2006. Functional sialidase residues R98, D131, R132, R205, E257, R273, R348 and Y382 (N1 numbering) are in cyan and framework sites E99, R136, W159, S160, D179, I203, E208, H255, E258 and E405 are in yellow. A46D is located in the stalk domain and is not visible in this model (B). Virus replication assays in primary chicken embryo kidney cells (CEK) (C) and primary normal human bronchial epithelial cells (NHBE) (D) infected at an MOI of 0.001 for indicated time points were done in three independent replicates. Results are shown as mean and standard deviation. Asterisks refer to statistical significance compared to rg-AL at p < 0.05 (see main text for details). Dashed lines in panels C and D indicate the predicted detection limit of plaque and foci assays in this study.

Sequence analysis of Egyptian H5N1 viruses (n = 509) isolated from humans (n = 126) and poultry (n = 383) from 2006 to 2016 revealed a particularly high prevalence of four missense mutations, i.e. A46D, L204M, S319F and S430G (N2 numbering equivalents: 48, 224, 339 and 451), in human-origin viruses compared to viruses from poultry (p < 0.005) (Fig 1A). Interestingly, we rarely observed these mutations individually, except for S319F in 2006–2007. Viruses with all four NA- mutations dominated after 2008 (HL-08) and those isolated in 2015–2016 (HL-16) possessed twelve novel NA mutations. Analysis of NA-N1 genes from poultry (n = 3311) and humans (n = 324) in Asia from 1997 to 2016 demonstrated low prevalence of these markers (Fig 1A), indicating enrichment of these mutations in human-H5N1 viruses in Egypt, where the highest number of H5N1-human infections are reported. Analysis of non-H5N1 sequences revealed 100% conservation of L204, except for influenza viruses of H8 subtype, which carry M204. A46D resides in the stalk domain, while the other mutations are in the head domain (Fig 1B). S319F is located in an antigenic site [19], while L204M resides between the sialidase catalytic and framework sites [20]. S430G is not part of any particular site hitherto identified. Molecular docking predicted that L204M alone or in combination with the other three mutations might decrease the binding of NA to the SA ligand (S1 Fig).

Based on these analyses, we generated seven recombinant viruses using Egyptian HL-H5N1 virus isolated in 2016 (designated rg-HL-16) as backbone. HL-16 is an avian isolate with 12 mutations that are found in isolates that have infected humans. Therefore, this isolate is used to represent human-isolates. In addition to rg-HL-16, six recombinant viruses carrying seven segments from rg-HL-16 and different NA segments were generated. These viruses include an H5N1 carrying NA resembling parent “avian-like” clade 2.2.1 (2006 virus, designated “rg-AL”), four viruses carrying avian-like-NA with single mutations designated as rg-AL-46D, rg-AL-204M, rg-AL-319F or rg-AL-430G, and an H5N1 carrying all four mutations resembling “human-like” 2.2.1.2 viruses from 2008 (rg-HL-08). None of the H5 viruses in this study was directly isolated from human cases.

### Single NA mutations reduced virus replication in avian, mammalian and human cell culture

Viruses carrying all four mutations (rg-HL-16 and rg-HL-08) or lacking all of them (rg-AL) replicated similarly in primary chicken cells (CEK) (p > 0.1) (Fig 1C) and canine MDCK-II (p > 0.1) or human A549 cell lines (p > 0.06) at 8 to 48 hpi (S2 Fig). Conversely, viruses carrying single mutations, particularly L204M, replicated less efficiently than rg-HL-16 and rg-AL (p < 0.05) in these cells, particularly at 8 hpi. In differentiated normal human bronchial epithelial (NHBE) cells, only three of the NA variants were tested: rg-AL, rg-HL-16 and rg-AL-204M. As controls, human (H1N1 and H3N2) and avian (H7N1) viruses were used (Fig 1D). Interestingly, after multiple cycle replication, rg-HL-16 replicated to significantly higher titers than human H1N1/H3N2 viruses and other recombinant H5N1 viruses tested in this experiment (p < 0.04). While the replication of rg-AL-204M was significantly lower than human H1N1/H3N2 viruses at 12 and 24 hours post-infection (hpi) (p < 0.05), they reached comparable titers 48 hpi. Together, the single NA mutations reduced H5N1 virus replication in different cells, with the lowest replication efficiency for rg-AL-204M. High replication efficiency in cell lines was restored by the combination of the mutations in human-like viruses (rg-HL-08 and rg-HL-16) or by avian-like viruses lacking these mutations (rg-AL), further indicating the interdependence and synergistic effect. In contrast, human-like H5N1 virus (rg-HL-16) replicated to higher levels than all other tested viruses in primary human airway epithelia.

### H5N1 viruses with human-like NA exhibited low NA activity conferred by L204M

**Fig 2:**
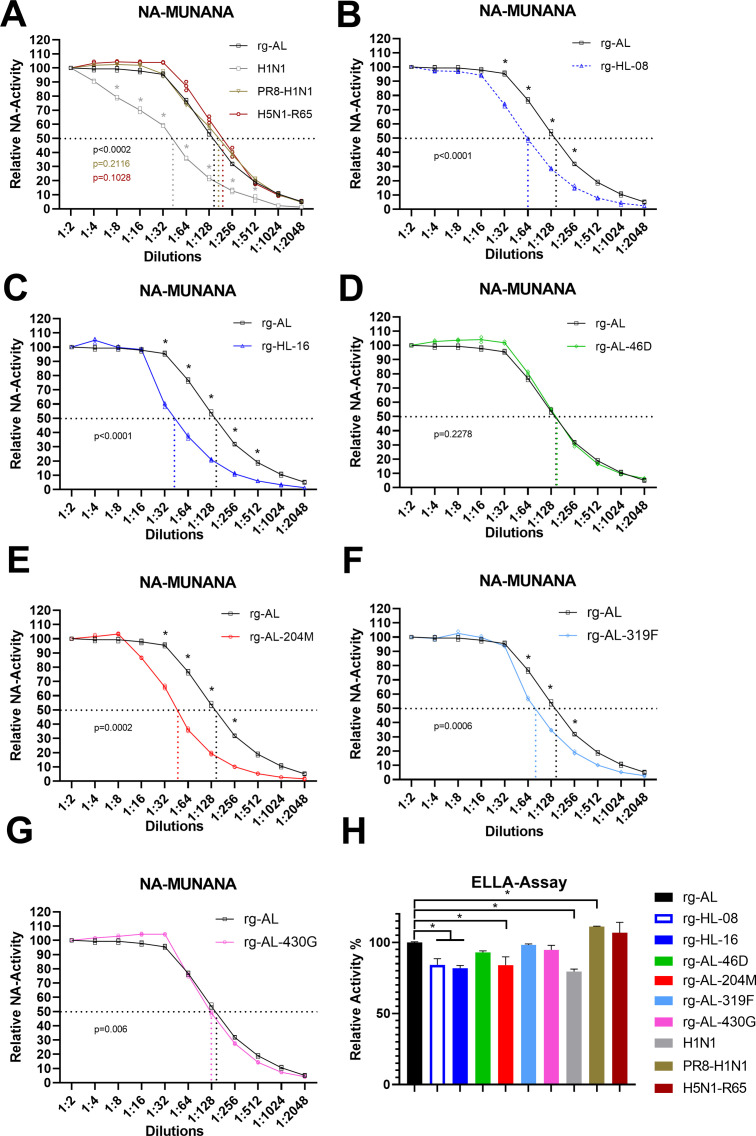
Impact of NA mutations on sialidase activity. Shown is the NA activity of Egyptian H5N1 viruses carrying different mutations against MUNANA (A-G) and fetuin (H). The experiments were done in three independent runs. The relative NA activity of different virus dilutions (y-axis) is compared to rg-AL (A-G). The p-values shown in the graphs refer to the statistical significance of the 50% NA-activity (shown as dashed lines) of indicated viruses compared to rg-AL (A-G). The activity of different viruses against fetuin in ELLA assay was calculated after adjustment of the absorbance (OD490) of rg-AL to 100%. The results are expressed as relative mean and standard deviation of three replicates (H). Asterisks refer to statistical compared to rg-AL at p value < 0.05. Viruses were adjusted to 32 HA units using chicken erythrocytes (and similar results were obtained after adjusting titers to plaque forming units).

We studied the impact of the mutations on NA activity by the fluorometric MUNANA assay and fetuin-based enzyme-linked lectin assay (ELLA) [21] (Fig 2). Human (H1N1), avian (H5N1/R65), and laboratory-adapted human H1N1-PR8 (PR8) were used as controls. Using MUNANA, rg-AL, H5N1/R65 and PR8 exhibited the highest NA activity, which was significantly higher than that of human-like H5N1 viruses (rg-HL-08 and rg-HL-16) (p < 0.05) (Fig 2A–2C). The NA activity of rg-HL-08 and rg-HL-16 are similar to human H1N1. While the introduction of L204M significantly reduced the NA activity of rg-AL to levels comparable to rg-HL-16 and rg-HL-08 (p < 0.0001) (Fig 2E). Although at lower levels than L204M, S319F reduced the NA activity of rg-AL (p < 0.002) (Fig 2F). A46D and S430G did not have a significant impact on the NA activity compared to rg-AL (p > 0.8) (Fig 2D and 2G). Using fetuin as substrate, similar patterns for L204M, rg-AL, rg-HL-08 and rg-HL-16 were observed (Fig 2H). Taken together, similar to human-H1N1 virus, human-like H5N1 viruses exhibited reduced NA activity conferred mainly by the L204M mutation.

**Fig 3:**
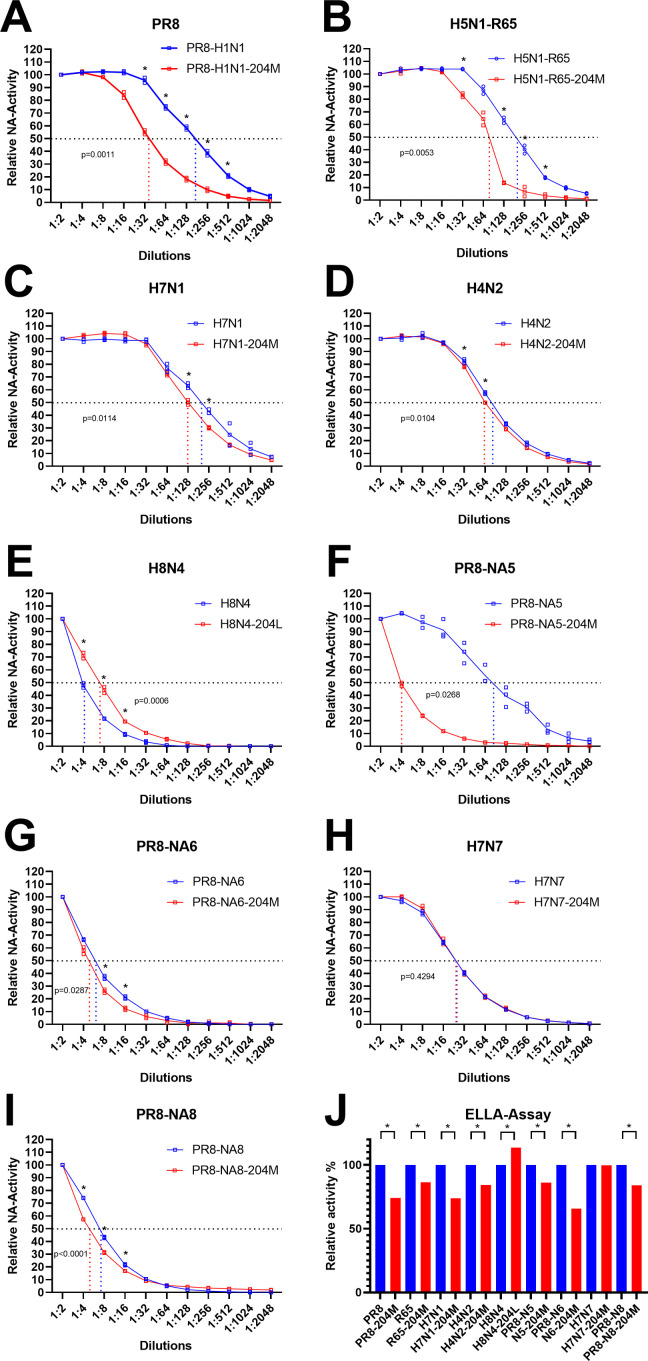
The impact of L204M mutation on NA activity of different subtypes of influenza viruses. Shown is the specific impact of L204M mutation on NA activity of different subtypes of influenza viruses carrying group 1 and group 2 NA against small substrate MUNANA (A-I) and large substrate fetuin (J). PR8-NA5, PR8-NA6 and PR8-NA8 were generated using seven gene segments from PR8 and NA from clade 2.3.4.4 H5N5, H5N6 or H5N8, respectively (see S1 Table). Viruses were adjusted to 32 HA units using chicken erythrocytes. The p-values shown in the graphs refer to the statistical significance of the 50% NA-activity (shown as dashed lines) of different 204-mutants compared to the wild-type viruses (A-I). The activity of different L204M-carrying viruses against fetuin in ELLA assay was calculated relative to the same virus dilution of the wild type viruses. Asterisks refer to statistical significance at p value < 0.05 as indicated (A-I) or compared to the NA activity of the wild type viruses in each subtype (J). ELLA-Assay was done in three independent runs and the results are expressed as mean and standard deviation of all replicates (black bars).

To address whether L204M can affect the sialidase activity of other IAV, the NA activity of nine recombinant IAV carrying NA of group 1 (N1, N4, N5, N8) and group 2 (N2, N6, N7) was determined (S1 Table). Insertion of L204M in different N1- as well as N5-, N6- or N8-type NA from contemporary panzootic H5Nx 2.3.4.4 viruses resulted in significantly reduced NA activity (Fig 3). A slight reduction, although still statistically significant (p < 0.01), in the NA activity of N2- and N7-type NA was also observed (Fig 3D and 3H). Interestingly, while H8N4 wild-type NA already carries M204, the reverse mutation L204 showed significantly increased NA activity (p < 0.0001) (Fig 3E). Using ELLA assay, all viruses carrying M204 exhibited significant lower NA activity except H7N7 (Fig 3J). Collectively, we found that L204M reduced the NA activity in several NA subtypes, including the recent H5Nx clade 2.3.4.4 viruses, indicating a broad effect across AIV subtypes.

### Low NA activity of human-like H5N1 is associated with reduced NA expression levels and amount of NA incorporated into virus particles

**Fig 4:**
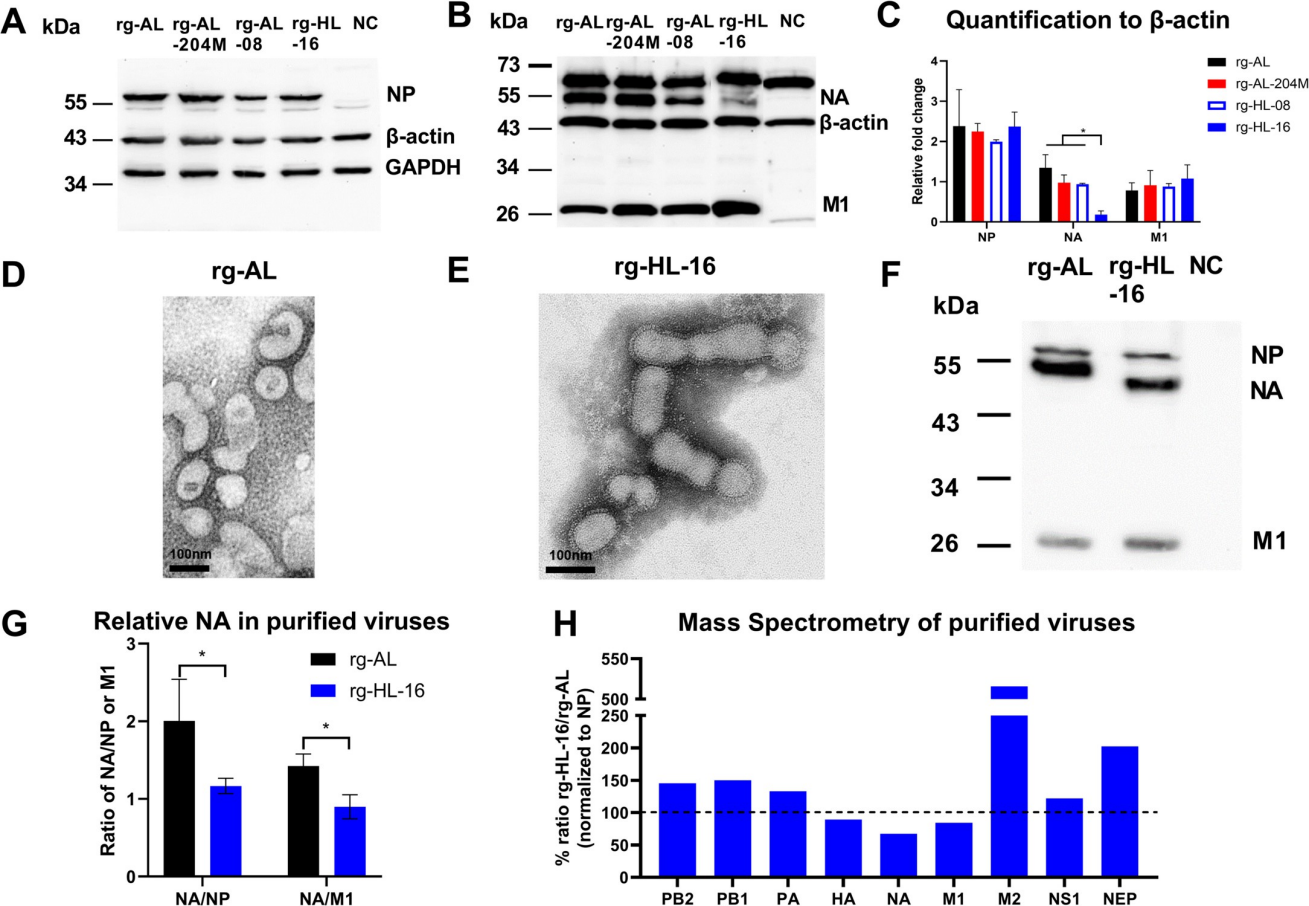
Relative NA expression in infected cells and purified virions. NA, M1 and NP expression in MDCK-II cells infected with an MOI of 1 with the indicated viruses for 24 h (A-C). Commercial monoclonal antibodies against M1, NP, β-actin or GAPDH (A) or polyclonal anti-N1-antibodies (B) were used as primary antibody. NC refers to non-infected cells as a negative control. The upper band in panel B is non-specific. The chemiluminescence signal of M1, NP or NA was normalized to β-actin (C). The test was done four times and the protein expression was calculated as mean and standard deviation of all replicates. Normalization of different proteins to β-actin or GAPDH yielded the same pattern (C). Shown is the ultrastructure of the purified rg-AL and rg-HL-16 viruses using negative staining (D, E) and the relative NA-content of purified virus particles using Western blot (F, G). Mass spectrometry-based quantification of viral proteins in purified virus particles. The amount of each protein of purified rg-AL and rg-HL-16 were normalized to the homologous NP protein. Relative results for rg-HL-16:rg-AL are shown (H). The results are representative for at least 3 independent experiments. The dashed line shows 100% expression level of rg-HL-16 in comparison to rg-AL. Asterisks indicate significant differences compared with rg-AL at p < 0.05.

To get an insight into the level of NA expression, we infected MDCK-II cells with viruses exhibiting high (rg-AL) or low NA activity (rg-AL-204M, rg-HL-08 or rg-HL-16) (Fig 4A and 4B). Using Western blot, we found that relative NP and M1 levels were comparable for all viruses. Regarding the NA, rg-AL expressed the highest NA amount. Insertion of L204M or the four combined mutations reduced the NA expression, although only marginally (p ≥ 0.058). The lowest NA level was found for rg-HL-16 (p < 0.005) (Fig 4A–4C). A similar pattern for reduction of NA levels was observed with other NA subtypes, except N7 (S3A and S3B Fig).

Interestingly, the reduction in the NA level of H5N1 viruses was not observed after transfection of avian DF1 cells (S3C and S3D Fig) or human A549 cells (S3E and S3F Fig) with pCAGGS-vector driven expression of the different NAs. The reduction in the NA levels probably requires the interaction of the NA gene with other gene segments. To further investigate this suggestion, we tried to rescue recombinant H7N7 virus (which was not affected by L204M mutation) carrying HL-16 NA alone or in combination with other HL-16 gene segments. All attempts failed to obtain H7N7 virus carrying HL-16 NA alone. Infectious H7 viruses were obtained when HL-16 NA was combined with HL-16 NP or M segments only, which shared the highest nucleotide sequence identity to the H7N7 NP and M segments (S2 Table). The NA expression after infection of MDCK-II cells with H7N7 carrying HL-16 NA plus M or NP was comparable to or higher than H7N7-NA (S3G and S3H Fig). These results indicate that the interaction with other gene segments (i.e. PB2, PB1, PA, HA and/or NS) play a role in the reduction of NA expression which remains to be studied.

Further, we analyzed whether the reduction in the NA activity may be attributed to the reduction of the amount of NA protein incorporated in purified virus particles. To this end, we propagated rg-AL and rg-HL-16 in embryonated chicken eggs (ECE) and confirmed the purity of viral preparations using transmission electron microscopy (Fig 4D and 4E). The relative amounts of NA protein were measured in purified virions using Western blot and mass spectrometry (Fig 4F–4H). Using Western blot, the relative amount of NA of rg-AL was significantly higher than rg-HL-16 after normalization to the NP or M1 proteins (p < 0.05) (Fig 4F and 4G). The results of mass spectrometry revealed an average reduction of ~32.7% of relative NA incorporated into rg-HL-16 compared to rg-AL (Fig 4H). Taken together, human-like H5N1 virus (rg-HL-16) demonstrated significantly reduced NA levels both in infected cells and the virions, which may explain the reduction in the NA activity of this virus.

**Fig 5:**
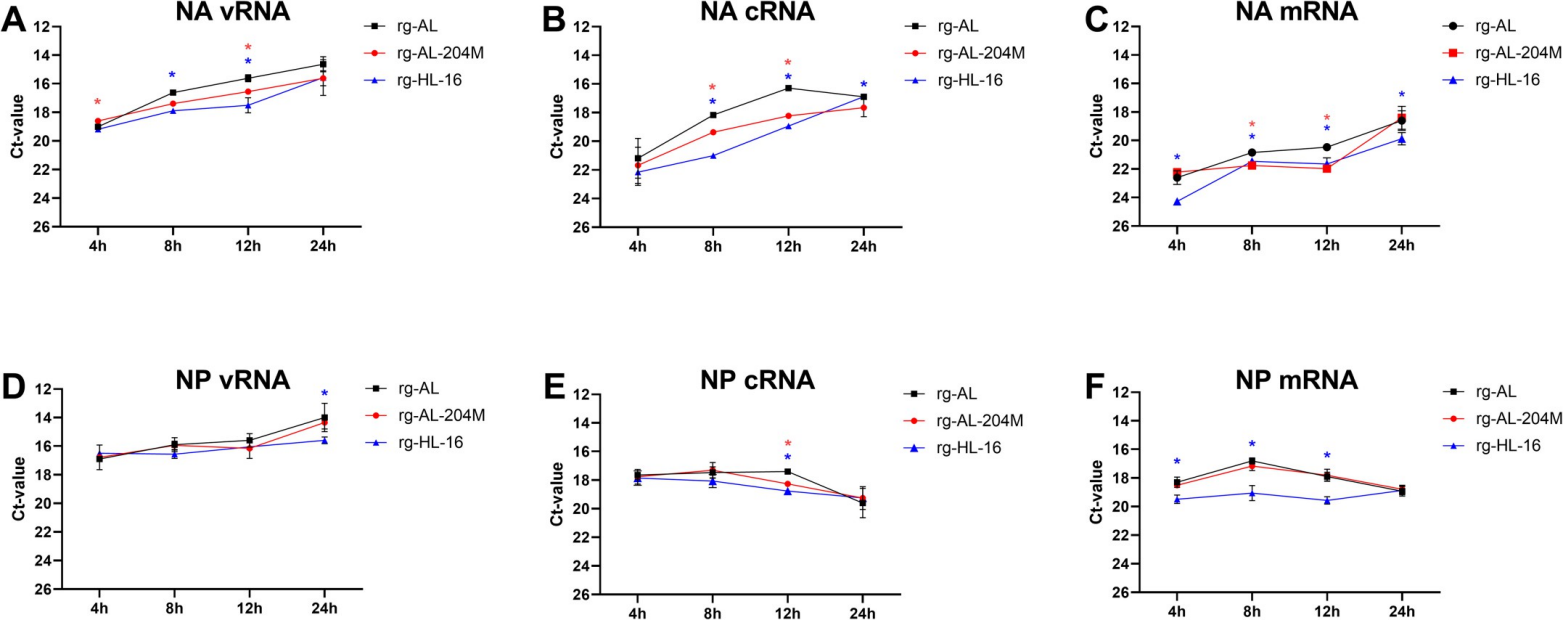
Quantification of NA vRNA, cRNA and mRNA in infected cells. Ct-values of strand specific RT-qPCR for detection of vRNA, cRNA and mRNA for NA (D-F) or NP (G-I) of indicated H5N1 viruses in MDCK-II cells infected with an MOI of 1 for 4, 8, 12 and 24 h. Results are shown as mean and standard deviation of two triplicate experiments. Asterisks indicate significant differences at p < 0.05.

To elucidate the mechanism for the reduction in NA expression, vRNA, cRNA and mRNA levels of the NA and NP segments of rg-AL, rg-AL-204M and rg-HL-16 at 4, 8, 12 and 24 hpi were determined by strand-specific RT-qPCR [22]. Compared to rg-AL, levels of NA vRNA and cRNA of rg-HL-16 were significantly lower at 8 and 12 hpi (p < 0.04), but not at 4 or 24 hpi (Fig 5A and 5B). Similarly, vRNA of rg-AL-204M was significantly lower than rg-AL at 8 hpi (p < 0.008) and cRNA levels were lower at 8 and 12 hpi (Fig 5A and 5B). Interestingly, rg-HL-16 exhibited significantly lower vRNA levels than rg-AL-204M at 4, 8 and 12 hpi (p < 0.04) (Fig 5A), while the NP vRNA and cRNA levels were almost comparable (p > 0.05) (Fig 5D and 5E). For mRNA, the levels of rg-HL-16 NA and NP mRNA were significantly lower than those of rg-AL at 4, 8 and 12 hpi (p < 0.001) and similar at 24 hpi (Fig 5C and 5F). Compared to rg-AL-204M, rg-HL-16 NA mRNA was lower at 4 hpi and NP mRNA at 4, 8 and 12 hpi (Fig 5C and 5F). Collectively, the reduction in the NA expression and incorporation in rg-HL-16 particles is likely due to reduced NA vRNA replication, which resulted in decreased cRNA synthesis and mRNA transcription.

To test whether the variable abundance of NA genes of rg-AL and rg-HL-16 can affect NA segment packaging, we transfected HEK293T cells with 9 plasmids, 8 full-set plasmids of rg-HL-16 and rg-AL NA. The co-transfection experiment was performed in three independent rounds by two different analysts after adjusting NA-plasmid concentration ratios to 1:1 (two rounds using two different stocks of NA plasmids) or 10:1 (third round). Plaques were selected after infecting MDCK-II cells and subjected to sequencing (S4 Fig). In the first round, 19/21 (90.5%) plaques revealed presence of the heterologous rg-AL NA, 1/21 (4.8%) contained the homologous rg-HL-16 NA and 1/21 (4.8%) carried both NAs. In the second round, 40/41 (97.6%) were positive for the heterologous rg-AL and 1/41 (2.4%) for both NA-types. In the third round, transfection of cells with 1 μg for rg-HL-16 NA and 0.1 μg for rg-AL NA revealed rg-AL NA in 5/14 (35.7%) and rg-HL-16 NA in 9/14 (64.3%) plaques. The results suggest that the heterologous rg-AL NA that produces more vRNA is preferentially selected for packaging over the homologous rg-HL-16 NA into reassortant H5N1 virus.

### L204M promoted plasma membrane accumulation of NA of human-like viruses and increased viral cell-to-cell spread

**Fig 6:**
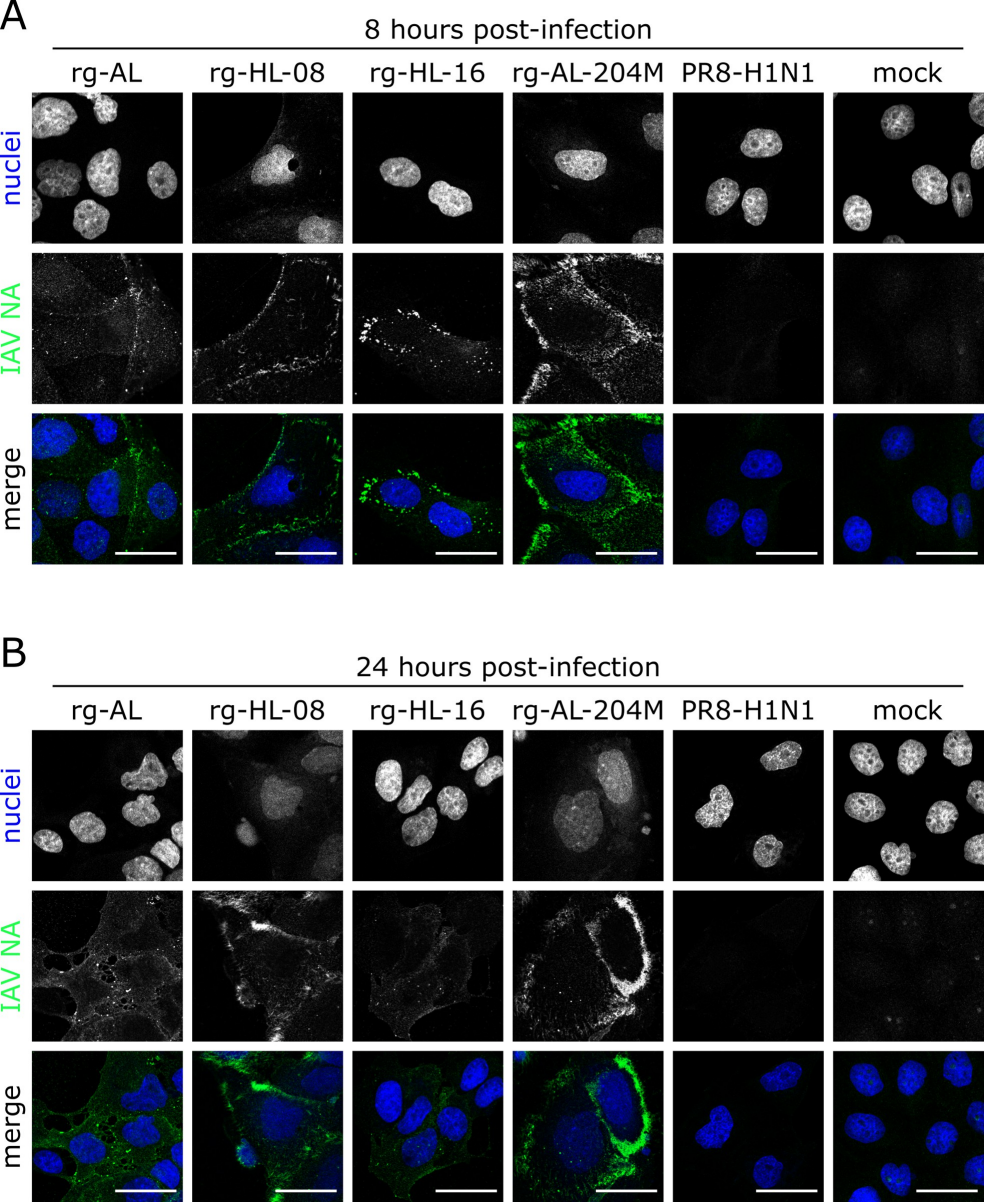
Cellular localization of NA in infected cells. Cellular localization of NA in infected MDCK-II cells without permeabilization. MDCK-II cells were infected with indicated viruses with an MOI of 1 for 8 (A) and 24 h (B). Cells were fixed and analyzed via confocal microscopy. Cell nuclei were counterstained with Hoechst33342. NA was detected by rabbit anti-N1 polyclonal antibodies. Confocal images were acquired with a Leica DMI6000 TCS SP5 confocal laser-scanning microscope (Leica Microsystems, Germany). Representative images of three independent experiments were processed using arivis vision4D (v3.4.0). Scale bar: 25 μm.

We further investigated the correlation between NA activity and virus distribution after infection of MDCK-II cells. Using confocal microscopy, we visualized the distribution of the NA in infected cells, 8 and 24 hpi with anti-N1 antibodies without permeabilization. rg-AL-204M and rg-HL-08 showed more abundant labelling on the plasma membrane than rg-HL-16, rg-AL and PR8 suggesting virus accumulation (Fig 6). These results suggested that the L204M has a role in the accumulation of human-like H5N1 NA on the cell membrane. Nonetheless, the effect of L204M could be compensated by other NA mutations as seen in rg-HL-16.

**Fig 7:**
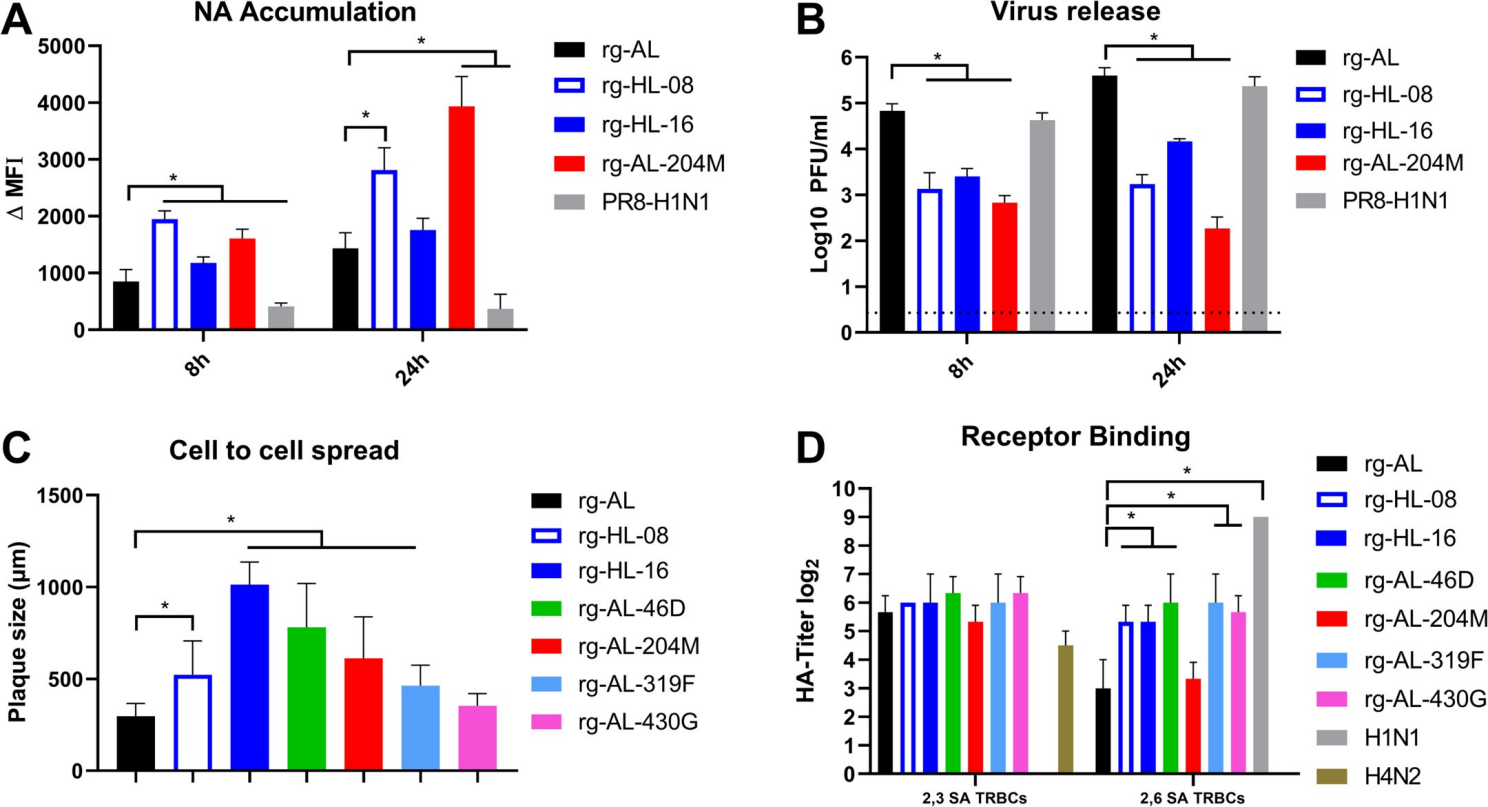
NA accumulation, virus release, cell-to-cell spread and affinity to modified turkey erythrocytes. MDCK-II cells were infected with the indicated viruses with an MOI of 1 for 8 and 24 h and the accumulation of NA on the plasma membrane was measured by flow cytometry and polyclonal anti-N1 antibodies. The results are the mean and standard deviation of three independent replicates (A). Virus release into the supernatant was determined by plaque test. The results are the mean and standard deviation of three independent replicates (B). Cell-to-cell spread of indicated viruses was assessed by measuring the size of 100 plaques induced in MDCK-II at 72 hpi using NIS Nikon Software (C). Viruses were adjusted to 32 HA units using chicken erythrocytes. Shown is the HA titer of indicated viruses against resialiated turkey erythrocytes carrying avian 2,3- or human 2,6-sialic acid (SA). The HA test was conducted in three independent experiments (D). Asterisks indicate significant differences compared with rg-AL at p < 0.05.

To confirm these results, we determined the relation between membrane bound NA and the amount of virus released into the supernatant of infected cells by flow cytometry and plaque test, respectively, at 8 and 24 hpi. As expected, accumulation of NA of rg-HL-16, rg-HL-08 and rg-AL-204M with low NA-activity on the plasma membrane was generally increased over rg-AL and PR8 with high NA-activity (p < 0.02) (Fig 7A). At 24hpi the amount of rg-AL-204M NA on the cell membrane was highest followed by rg-HL-08 and rg-HL-16 (p < 0.005). Notably, virus release as measured by plaque test inversely correlated with surface NA accumulation. Compared to PR8 and rg-AL, the titers of rg-AL-204M, rg-HL-08 and rg-HL-16 in the supernatant were significantly lower (p < 0.001) (Fig 7B). These data further suggest that L204M plays a role in virus release into the supernatant of infected cells possibly due to an accumulation on the plasma membrane.

To study whether NA accumulation on the plasma membrane can promote viral cell-to-cell viral spread, plaque diameters induced by the different viruses in MDCK-II cells were measured (Fig 7C). rg-HL-16 and rg-AL-204M produced significantly larger plaques than rg-AL (p < 0.0001). Likewise, also the other viruses, except rg-AL-430G, had significantly larger plaques than rg-AL (p < 0.0001). Together, human-like mutations in the NA (except S430G) alone or combined increased H5N1 cell-to-cell spread.

### NA mutations increased H5N1-affinity to human-type receptors

**Fig 8:**
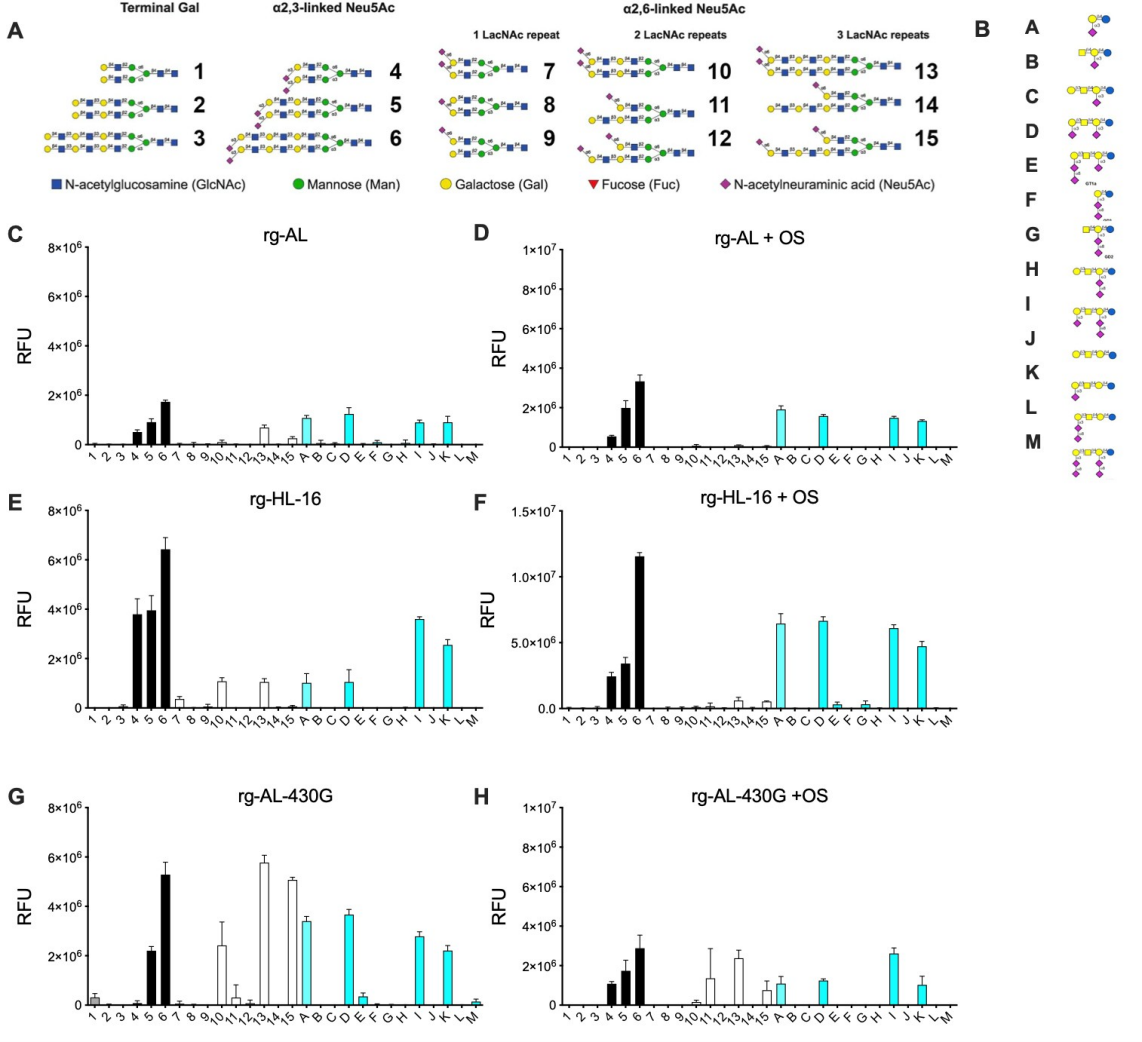
The impact of NA on binding specificity to avian- and human-like glycan moieties. Shown in numbers are N-glycan compounds terminating in gal (#1–3), avian type receptors (#4–6, black bars) and human type receptors (#7–15, white bars) (A). Glycolipids presentation of avian-type receptors are shown as #A-M magenta bars [25,80] (B). Viruses were applied to the array surface in the absence (C, E, G) or presence of oseltamivir (OS) (D, F, H) at concentration of 200 nM (n = 4). Representative results for three independent experiments are shown for rg-AL (C and D), rg-HL-16 (E and F) and rg-AL-430G (G and H).

Besides the HA, NA can also bind directly to cellular receptors affecting virus entry [23,24]. Therefore, we firstly studied the receptor binding affinity to turkey erythrocytes (TRBCs) expressing either avian 2,3-SA or human 2,6-SA in standard HA test [5]. As expected, human H1N1 bound efficiently to 2,6-SA but not 2,3-SA (p < 0.001), while avian H4N2 did not show affinity to 2,6-SA (Fig 7D). All H5N1 viruses showed comparable affinity to 2,3-SA (p = 0.9). However, human-like viruses (rg-HL-16 and rg-HL-08) exhibited ≥ four-fold higher binding affinity to 2,6-SA than rg-AL and rg-AL-204M (p < 0.04). Insertion of A46D, S319F or S430G into rg-AL significantly increased binding-affinity to 2,6-SA (p < 0.01). Interestingly, only rg-AL-319F and rg-AL-430G had similar binding-affinity to 2,3-SA and 2,6-SA. To confirm these results, we tested the receptor specificity of all H5N1 viruses using a glycan microarray presenting preferred receptors for human viruses [25]. The viruses were screened on the array with and without oseltamivir (OS) to disentangle HA specificity and the influence of NA on receptor binding. All viruses bound to avian-like 2,3-linked Neu5Ac glycans (Fig 8) overall signal was decreased without OS indicated contributions of NA. rg-AL fails to convincingly bind human-type receptors, which was even more clear in the presence of OS, however, indicating that this NA might has a sialic acid binding function. For rg-HL-16 bound to some human-like 2,6-linked Neu5Ac was observed in the absence of OS (Fig 8). Interestingly, the shift in binding specificity of rg-HL-16 was probably due to NA S430G (Fig 8). NA S430G was especially interesting as it binds human-type receptors in both the absence and presence of OS, while the HA matches the other viruses. Thus, the enzymatic site is not responsible for the binding to the human-like 2,6-linked Neu5Ac. In summary, human-like viruses (rg-HL-16 and rg-HL-08) had dual binding affinity to avian- and human-type receptors, and NA S430G mutation plays a role in the affinity to human-type receptors.

### NA retained specificity and cleavage of avian-type sialic acid receptors during virus entry

The release of human-like virus from infected cells can be also affected by the density of sialic acid on the cell surface. To explore the efficiency and specificity of NA to remove SA-receptors during virus entry, an underappreciated role of the NA, we infected MDCK-II cells which contain both 2,6-SA and 2,3-SA, and genetically modified MDCK-SIAT1 cells, which express elevated amount of 2,6-SA and reduced amount of 2,3-SA [26]. Cells were infected with rg-HL-16 and rg-AL for two hours only and the amount of 2,3-SA and 2,6-SA was measured using flow cytometry (S5 Fig). Compared to non-infected cells, we found that both viruses were efficient in removal of 2,3-SA (p < 0.0001). Interestingly, in MDCK-II, but not MDCK-SIAT1, cells rg-AL was more efficient than rg-HL-16 to cleave 2,3-SA and 2,6-SA (p < 0.03). These results indicate that NA retained specificity to cleave avian-type receptors during entry, which is probably essential in reducing the density of SA bound to the HA at late stage of release. The presence of high amount of 2,6-SA on cell surface may further explain the inability of rg-HL-16, which has a higher affinity/specificity to this receptor, to be efficiently released compared to rg-AL.

### Altered NA activity did not affect the tropism of H5N1 viruses to non-ciliated airway epithelia

Previous studies showed that some human influenza viruses preferred ciliated epithelial cells (ECs) with both 2,3- and 2,6-SA, while an AIV H5N1 preferentially infected non-ciliated mucin-producing goblet cells rich in 2,3-SA [27, 28]. To determine whether Egyptian human-like H5N1 viruses attach either to ciliated or non-ciliated epithelial cells and whether NA mutations alter viral airway epithelia tropism, differentiated primary ferret airway ECs (FAECs) were infected with rg-AL, rg-AL-204M, rg-HL-16 or human H3N2 (S6 Fig). All H5N1 viruses infected the FAECs and were mainly detectable in non-ciliated ECs. Conversely, human H3N2 infected efficiently both ciliated and non-ciliated cells. These results indicate that Egyptian H5N1 viruses can infect cells resembling the respiratory tracts of mammals, but less efficiently than human H3N2 virus. Altered NA activity apparently does not affect tropism to the airway epithelia.

### NA mutations affected H5N1 virulence in mice but not in ferrets or chickens

To assess the impact of the NA mutations on the virulence *in vivo* we infected chickens, mice and ferrets with the different viruses. Chickens were inoculated intranasally (IN) with H5N1 viruses, and 1-day post inoculation (dpi) naïve chickens were added to assess virus transmission (Table 1). All IN-inoculated chickens and their contacts died within 3 dpi and 4 days post-contact, indicating no role for NA mutations in virulence or transmission in chickens.

**Table 1:** Virulence of recombinant H5N1 viruses in chickens after intranasal inoculation.

Virus	Inoculated chickens			Contact chickens	
	Mortality	PI	MTD	Mortality	MTD
rg-AL	6/6	2.5	2 days	4/4	4.5 days
rg-HL-08	6/6	2.5	2 days	4/4	4.0 days
rg-HL-16	6/6	2.4	2 days	4/4	5.0 days
rg-AL-46D	6/6	2.4	3 days	4/4	4.5 days
rg-AL-204M	6/6	2.3	3 days	4/4	5.0 days
rg-AL-319F	6/6	2.3	3 days	4/4	5.0 days
rg-AL-430G	6/6	2.4	2.8 days	4/4	5.0 days

Chickens were inoculated intranasal with 10^4^ PFU/bird and one-day post infection (dpi) naïve chickens were added to each group to assess virus transmission.

PI = pathogenicity index; it was calculated as mean value of daily clinical scoring of animals as 0 (healthy), 1 (showing one clinical sign: depression, cyanosis of shanks, comb and wattles, respiratory distress, diarrhoea), 2 (showing two clinical signs) and 3 (dead) for 10 days according to the OIE guidelines. MTD = mean time to death (days after inoculation).

**Fig 9:**
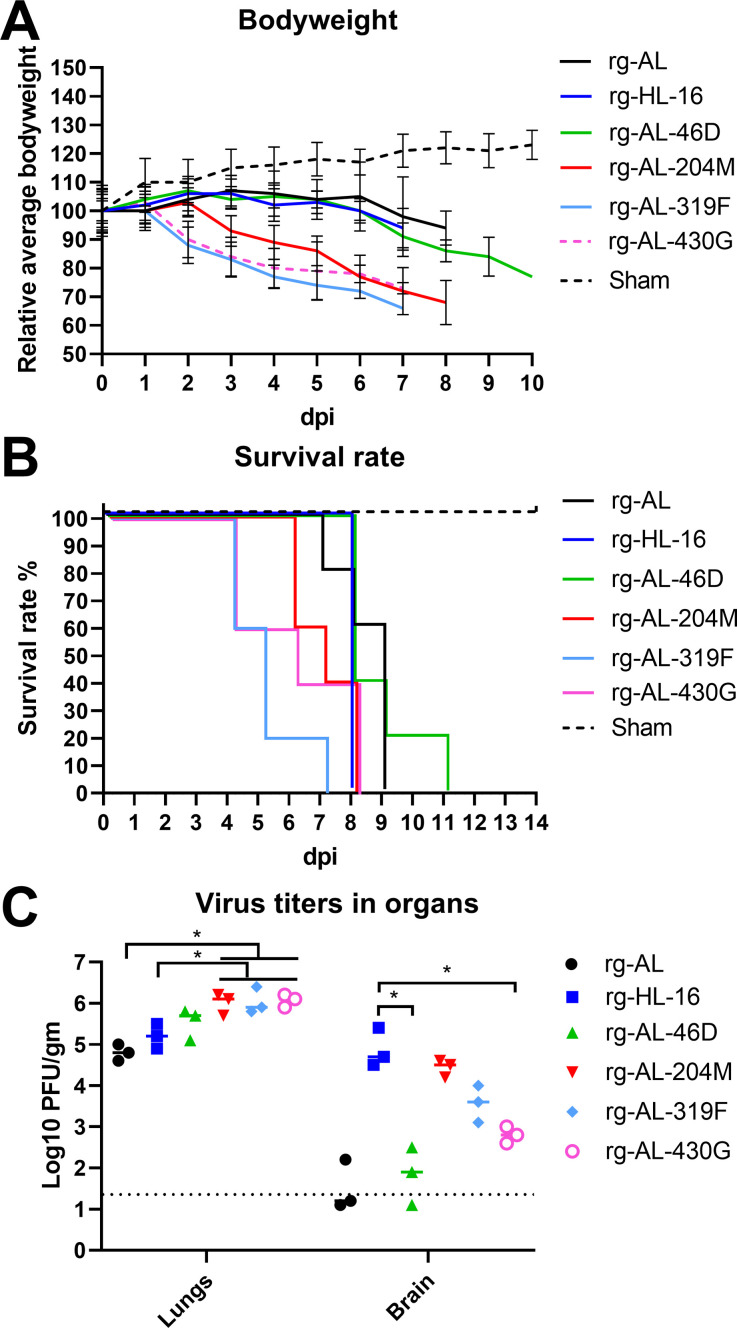
The impact of NA mutations on virus virulence in mice. Mice (n = 8 per group) were inoculated intranasally with 10^5^ PFU of the indicated viruses or 50 μL PBS (sham group). Average bodyweight (A) and the survival curve (B) were determined. Viral loads in lung and brain homogenates were tested by plaque test and were expressed as PFU/gram (n = 3 per group) (C).

We further assessed the reduction in bodyweight (BW), mean time to death (MTD), viral replication and early immune response in mice inoculated IN with H5N1 viruses. Compared to the PBS-inoculated (sham) group, all inoculated mice exhibited significant BW loss (p < 0.002) (Fig 9A). A46D did not significantly affect virulence or replication of rg-AL in mice. Viruses lacking any of (rg-AL) or carrying all 4 mutations (rg-HL-16) induced similar levels of BW loss (p > 0.8), while single insertion of S319F, S430G and L204M caused a significant reduction in BW compared to other viruses (p < 0.04). Infected mice in these groups presented with neurological signs. All infected mice died within 11 days (Fig 9B). Similarly, viruses lacking (rg-AL) or carrying all 4 mutations (rg-HL-16) had comparable MTD (8.4±0.4 day), while mice inoculated with rg-AL-319F, rg-AL-430G or rg-AL-204M died earlier (MTD 5 to 7 days) (p < 0.003). Infectious viruses were isolated 3 dpi from lungs of all euthanized mice (Fig 9C). While animals infected with rg-AL and rg-HL-16 had similar virus titers in the lungs, rg-AL-204M, rg-AL-319F and rg-AL-430G infected animals exhibited significantly higher titers (p < 0.05). Both, rg-AL and rg-AL-46D were isolated from the brain of 1/3 or 2/3 mice, respectively, at lower titers than other viruses. rg-HL-16 and rg-AL-204M had the highest titers followed by rg-AL-319F then rg-AL-430G and were found in the brain of all mice (n = 3) (Fig 9C). To study the impact of NA mutations on the early immune response, activation status of CD4^+^ and CD8^+^ T-cells was measured by flow cytometry 3 dpi in the spleen (S7 Fig). Compared to rg-AL, CD4^+^ (but not CD8^+^) were activated to significantly higher levels in mice inoculated with rg-AL-204M and rg-AL-319F (p < 0.05). Collectively, viruses with (rg-HL-16) or lacking (rg-AL) these four mutations exhibited a comparably moderate virulence in mice, while viruses with single mutations in the head domain displayed higher virulence.

To assess virulence and transmission in ferrets, two ferrets per group were inoculated IN with rg-AL, rg-AL-204M, rg-HL-16 and human H3N2. Two naïve ferrets were added 1 dpi to assess virus transmission. H3N2-inoculated and contact ferrets exhibited moderate (score 2) to severe (score 3) flu-like illness (S8A Fig and S3 Table). Clinical signs caused by rg-AL and rg-HL-16 were more severe than those induced by rg-AL-204M but less than observed in H3N2 infected animals. Moreover, viral RNA in nasal washes was detected in all inoculated ferrets (S8B-C). H5N1 viruses were found at significantly lower levels than H3N2 in inoculated ferrets at 2 dpi only (p < 0.0001). Both H3N2 sentinels excreted viruses at 2–10 dpi. One rg-HL-16 sentinel shed virus 4 dpi, at a lower level (10^1.4^ PFU/ml) than the H3N2 sentinels (10^3.7^ PFU/ml). Trials to measure infectious virus titers in plaque test failed. NP-antibodies were detected in all inoculated ferrets and only in 1/2 and 2/2 sentinels cohoused with rg-HL-16- and H3N2-inoculated ferrets, respectively (S3 Table). Together, all tested Egyptian H5N1 viruses can replicate and cause disease in inoculated ferrets but at lower levels than human H3N2 virus. Mutations in the NA did not affect severity of clinical signs and the poor transmission of the H5N1 viruses in this study.

## Discussion

Bird-to-human transmission of AIV including H5Nx Goose/Guangdong poses a serious zoonotic and pandemic threat. It is important to understand the genetic markers for efficient replication of these viruses in human cells. Here, we describe the prevalence and the role of mutations in the NA preferentially selected in human H5N1 viruses. Compared to their avian ancestors, we show here that human-like H5N1 viruses exhibit a significantly lower NA activity, which is similar to human influenza viruses exhibit low NA activity [29]. Hence, the HA of human IAV has generally less affinity for 2,6-SA than HA of AIV for 2,3-SA [30], it is conceivable that the NA of human viruses exhibits reduced activity to optimize the receptor binding/destroying balance [31]. Previous studies suggested that reduced NA activity of AIV might be required for the adaptation of NAs of AIV (e.g. H5N1, H9N2) to growth in human cells [29, 32]). Moreover, variable physiological and anatomical differences (e.g. mucin [33, 34] or SA distribution [35]) in poultry and humans might play a role in the selection of viruses with reduced NA activity in humans. Here, the reduction of the NA activity of human-like H5N1 viruses (conferred partially by L204M) is not only due potential conformational changes in the NA, as predicted in this study [29, 36] but most likely due to the low amount of NA incorporated into virions. Similar to our results, Das et al. [37] found that mutations in the NA (e.g. G357S) in PR8 reduced the relative amount of NA incorporated into virus particles.

The molecular mechanism underlying reduced expression and incorporation of NA into virus particles has not been completely clarified, particularly for the current zoonotic AIV. Our transfection experiments in avian and human cells and replacing H7N7 NA with H5N1 NA did not result in reduced NA expression. We also found that the level of RNA replication and transcription of human-like NA was lower than that of avian NA RNA, and the abundant avian NA was selected over the homologous human-like NA in the co-transfection experiments. These results suggest that reduced vRNA, cRNA, mRNA and NA expression (and probably packaging) of human-like viruses is affected by the interaction with other gene segments. It has been previously shown that the interaction or co-segregation of NA with NP or PB1 affected the packaging of NA and subsequently viral fitness [38, 39]. Two different studies have shown that the HA/NA ratio of human influenza viruses is strain-dependent [40] and that this ratio can be affected by the reassortment with other gene segments, particularly PB1 [41].

The role of NA in receptor binding has been frequently reported. Similar to previous results [23, 24], we found that mutations in the NA of human-like H5N1 viruses play a role in binding to human-like receptors. The NA of some AIV has a second SA-binding site (2SBS) formed by three peptide loops, 370 loop (residues 366–373, N2 numbering), 400 loop (residues 399–404), and 430 loop (residues 430–433) with direct SIA contact residues: S367, S370, S372, N400, W403, and K432 [42, 43]. The zoonotic H7N9 virus in China in 2013 exhibited enhanced binding to human-type receptors, which in part conferred by the 2SBS and the sialidase sites [44]. Our experiments using glycan microarray showed that the binding of NA to human-type like receptors, conferred mainly by S430G, is probably independent of the sialidase pocket. Additional experiments are required to determine whether these four human-like H5N1 mutations induce conformational changes in the 2SBS [42, 45]. Importantly, it has been argued that the binding of NA to the SA-receptor rather than the catalytic cleavage activity may be advantageous for the initial entry of zoonotic influenza viruses on the expense of virus release, which may be a limiting factor in the transmission efficiency of these viruses *in vivo* [44]. Indeed, during virus entry, we found that NA removed the 2,3-SA and to a lesser extent, if at all, 2,6-SA. This reduction in avian-type receptors during virus entry might reduce adherence of the HA of progeny virions to 2,3-SA during virus release. It is also tempting to speculate that the presence of high amounts of 2,6-SA during virus release may be the main driver for adaptation of AIV HA and probably NA to human-type receptors. Further experiments should be conducted to elucidate this hypothesis.

Notably, there was a clear synergism and interdependence between these four co-evolving mutations to maintain high replication efficiency *in vitro* and to confer moderate virulence and longer survival rate in mammals without affecting fitness in chickens. Similarly, 4 substitutions (N308S, A346V, T442A and P458S) in the NA of the zoonotic H7N7 viruses isolated during the large outbreak in the Netherlands in 2003 were described, which were not part of any known NA domain [46]. These mutations synergistically affected NA activity, virus replication and release [46]. Similar to our findings, single mutations, particularly in the head domain, increased mortality in H1N1-infected mice due to severe neurological disorders [47] or probably via early activation of strong immune response and/or increased replication in the lungs [47–49]. Furthermore, we observed that the binding-affinity to 2,3-SA was generally higher than to 2,6-SA as previously reported [15, 50, 51]. This might explain the distinct tropism of H5N1 to non-ciliated cells we observed in our fully-differentiated FAECs, as 2,3-SA has been demonstrated to be exclusively present on non-ciliated cells in differentiated primary ferret epithelial cell cultures [51]. Moreover, comparing results of early (rg-HL-08) and late (rg-HL-16) human-like H5N1 viruses indicated a progressive reduction in the NA expression and increased cell-to-cell spread. However, it is not clear how enhanced cell-to-cell spread *in vitro* can be advantageous, if at all, to the spread of the virus (with low NA activity) in the mucus producing cells in the airway epithelia *in vivo* where the virus is trapped [52].

Although Egypt reported the highest number of H5N1-human infections, the case-fatality rate was remarkably low [18]. In a recent study, Egyptian human-like H5N1 viruses replicated at modest levels in inoculated ferrets and aerosol transmission was observed in some experiments [50]. Here, human-like H5N1 was less virulent and transmissible in ferrets than human H3N2 and the NA mutations did not dramatically affect the H5N1 fitness. It is worth mentioning that H5N1 in our study has a NA-stalk deletion, which is commonly seen in poultry-adapted viruses [5, 53]. Shortening the H1N1-NA stalk domain reduced the NA activity and correlated with poor direct-transmission in ferrets [34, 54]. Similarly, trapping of AIV in goblet cells correlates with the low transmissibility of AIV in ferrets [52]. Therefore, it is possible that Egyptian H5N1 viruses require additional mutations in the HA e.g. to increase affinity to 2,6-SA and reduce affinity to 2,3-SA, in the viral polymerase (e.g. PB2-D701N) and/or M1/M2 proteins to transmit efficiently from ferret-to-ferret [55–57].

In conclusion, human-like H5N1 strains had acquired four NA mutations, which act synergistically to keep the balance between virus replication and moderate virulence in mammals. Importantly, these mutations reduced NA activity, expression and incorporation into virions, and enhanced virus binding affinity to human-type receptors. The NA played a role in viral replication in human airway epithelial cells and in mice without affecting fitness in chickens. Therefore, mutations in the NA accompanied the transmission of AIV to humans should be carefully monitored. Hence, vigilance to regularly assess replication of AIV in mammals is highly warranted to react early against emerging viruses with pandemic potential.

## Materials and methods

### Ethics statement

Animal experiments were conducted following the German Regulations for Animal Welfare after approval by the authorized ethics committee (Landesamt für Landwirtschaft, Lebensmittelsicherheit und Fischerei Mecklenburg-Vorpommern LALLF M-V, approval number 7221.3–1.1-051/12 and 7221.3-1-060/17) and the commissioner for animal welfare at the Friedrich-Loeffler-Institut (FLI) representing the Institutional Animal Care and Use Committee.

### Viruses and cells

All viruses used in this study are listed in S1 Table. Human-like clade 2.2.1.2 A/turkey/Egypt/AR1507/2016 (H5N1) (HL-16) was isolated in Egypt [58]. A/quail/California/D113023808/2012 (H4N2) was kindly provided by Beate Crossly, UC-Davis. A/Giessen/8/2018 (H1N1) and A/Victoria/3/1975 (H3N2) was obtained from the repository of Institute of Medical Virology, Giessen. Recombinant A/PR/8/34 (H1N1) was kindly provided by Jürgen Stech from Friedrich-Loeffler-Institut, Riems, Germany (FLI). A/swan/Germany/R65/2006 (H5N1), A/chicken/Italy/473/1999 (H7N1), A/chicken/Germany/R1361/2011 (H7N7), A/turkey/Germany-SH/R425/2017 (H5N5), A/common pochard/Germany-BY/AR09-18-L02421/2017 (H5N6), A/tufted duck/Germany/8444/2016 (H5N8) and A/turkey/Ontario/6118/1968 (H8N4) viruses were kindly provided by Timm C. Harder, FLI (S1 Table). MDCK-SIAT1 cells were obtained from the Institute of Virology, Philipps University, Marburg [26]. Other cell lines in this study were obtained from the cell-culture collection at the Veterinary Medicine of the FLI. CEK cells were prepared according to the standard protocols. NHBE cells were prepared and differentiated according to the manufacturers’ recommendations (Lonza).

### Differentiated FAECs

Primary FAECs were isolated from the airways of one-year-old ferrets, modified from previous descriptions for other species [59, 60]. Essentially, after the lung had been removed from the thoracic cavity, the proximal airways were dissected, washed with phosphate-buffered saline (PBS), and digested in Dulbecco’s Modified Eagle’s Medium (DMEM, Gibco; Thermo Fisher, USA) supplemented with 1 mg/mL pronase (Roche, Switzerland), 10 μg/mL DNase I (AppliChem, Germany), 100 U/mL penicillin (Sigma-Aldrich, Germany), 100 μg/mL streptomycin (Sigma-Aldrich), 50 μg/mL gentamicin (Gibco; Thermo Fisher, USA), 1.25 μg/mL amphotericin B (Sigma-Aldrich), and 2 μg/mL fluconazol (Claris Lifesciences, UK) for 48 h at 4°C with gentle shaking. Primary FAECs were harvested by scraping the cells from the inside of the digested bronchi with a scalpel (#10). The cells were expanded in collagen type I (PureCol; Advanced BioMatrix, USA)-coated cell culture flasks with PneumaCult-Ex medium (STEMCELL Technologies, Canada) at 37°C and 5% CO2 until they reached 70–90% confluency at which point 5 × 10^4^ cells per insert were transferred to collagen IV (Sigma-Aldrich)-coated cell culture inserts (0.33 μm^2^, polyethylenterephtalate [PET] membrane, 0.4 μm pore size; Corning, USA). The cells were kept submerged in PneumaCult-Ex medium until they reached confluency. Subsequently, FAECs were airlifted by removing PneumaCult-Ex medium from the apical compartment and switching the medium in the basal compartment to PneumaCult-ALI medium (STEMCELL Technologies). To achieve full mucociliary differentiation, the cells were cultured under air-liquid interface (ALI) conditions for four weeks at 37°C and 5% CO2. ALI medium in the basal compartment was exchanged every 2–3 days and the cell surface was washed once per week with PBS. Infections of fully-differentiated FAECs were each performed in duplicate on cells originating from three independent donor ferrets (n = 6 per virus). To this end, the apical surface of the FAECs was washed three times with PBS and inoculated with 5 × 10^5^ PFU. For mock-infected controls, ALI medium was used. Following a 2 h-incubation at 37°C and 5% CO2, inoculum was aspirated and the cell surface was washed twice with PBS. Infected FAECs were kept under ALI conditions until the end of the experiment.

### Generation of recombinant viruses

Cloning of gene segments of rg-HL-16 and rescue of recombinant viruses were done as previously described [61]. All mutagenesis assays were conducted using standard QuikChange II XL Site-Directed Mutagenesis Kit (Agilent Technologies, USA). The recombinant viruses were propagated in 9–11 day-old specific pathogen free (SPF) embryonated chicken eggs (ECEs) [62], except PR8 and H3N2 which were propagated on A549 cells. Viral titers of working stocks were determined by plaque assay as described below.

### Replication kinetics and plaque test

Growth kinetics were done at an MOI of 0.001 and cells were harvested at the indicated time points. Virus titers were quantified by ten-fold serial dilution in minimal essential media (MEM) containing bovine serum albumin (BSA) and infection of MDCK-II cells in plaque assay or focus forming assay (FFA) (only NHBE). N-tosyl-L-phenyalanine chloromethyl ketone (TPCK)-trypsin was used for replication and titration of low pathogenic avian influenza viruses (H4N2, H7N1, H7N7, H8N4) and human influenza viruses (PR8, H1N1 and H3N2). The replication kinetics were conducted in duplicates and repeated three times. The results are expressed as the mean and standard deviation of all replicates. Cell-to-cell spread was determined by measuring plaque size in MDCK-II cells as previously described [63].

### NA activity assays

2’-(4-Methylumbelliferyl)-α-D-N-acetylneuraminic acid (MUNANA)-based neuraminidase activity assay was done using NA-Fluor Influenza Neuraminidase Assay Kit (Applied Biosystems, USA) according to the manufacturer’s guidelines and results were measured using Infinite M200 PRO (Tecan). Briefly, virus suspensions were adjusted to 32 HA units (or 10^5^ pfu/ml) using chicken erythrocytes. Fifty microliters of each virus, in two different wells, were two-fold serially diluted in 50 μL 1X assay buffer in Thermo Scientific NUNC 96-well black flat bottom plates and 50 μL of 200 μM NA-Fluor substrate working solution was added to each well. After one hour at 37°C, the reaction was terminated by the addition of 100 μL of NA-Fluor stop solution and the fluorescence was measured with an excitation wavelength range of 350 nm to 365 nm and an emission wavelength range of 440 nm to 460 nm in Infinite M200 PRO (Tecan, Germany). After subtraction of the reading background value, virus dilutions were plotted versus the average of the relative fluorescence unit (RFU). The assay was repeated in three independent runs and results are shown as the mean and standard deviation of all replicates. The NA activity was also determined by enzyme-linked lectin assay (ELLA) as described with few modification [21]. Briefly, 96-well plates were coated for 18 hr with 25μg/mL Fetuin 100μL/well then washed three times with PBST. Viruses were adjusted to 32 HA units using chicken erythrocytes. A total of 24μL of each virus, in four different wells, was added to 216μL sample diluent and incubated at 37°C for up to 18 hr. Plates were washed 6X with PBST and 100 μl/well PNA-HRPO solution was added. After 2 hr at room temperature, plates were washed 3 times to remove the PNA- HRPO and 100 μl of the O-phenylenediamine dihydrochloride OPD (Sigma-Aldrich, Germany) substrate was added to each well for 10 minutes at room temperature in the dark before adding 100 μl/well of 1N sulfuric acid to stop the reactions. The Optical Density (OD) of all the test plates was read at 490 nm for 0.1 sec using Infinite M200 PRO (Tecan, Germany). The background values were subtracted from values of reads of wells containing viruses, then values of wild type viruses were set at 100%. The values of different mutants were presented relative to that of the wild type viruses. The assay was repeated three times and results are the mean and standard deviation of all replicates.

### Receptor binding specificity assays

The receptor binding specificity of all indicated viruses to avian α(2,3)- or mammalian α(2,6)-linked sialic acid moieties or glycans was done using modified TRBCs as described [55] and glycan microarray as previously published [25]. For the HA test, viruses were firstly adjusted to 32 HA units using chicken erythrocytes. Resialylation of TRBCs was done using α2,6-(N)-sialyltransferase (Takara) or α2,3-(N)-sialyltransferase (Sigma Aldrich) and CMP-sialic acid (Sigma-Aldrich). Results of three intendent triplicate-experiments are expressed as the mean of all replicates. For the glycan microarray studies, the printed library of compounds comprised the glycans and quality control procedures were published previously [25]. Virus isolates (25 μL) were diluted with PBS-T (PBS + 0.1% Tween, 25 μL) and applied to the array surface in the presence of oseltamivir (200 nM) (Sigma Aldrich) in a humidified chamber for 1 h, followed by successive rinsing with PBS-T (PBS + 0.1% Tween), PBS and deionized water (2x) and dried by centrifugation. The virus-bound slide was incubated for 1 h with the CR6261 influenza hemagglutinin stem-specific antibody (100 μL, 5 μg mL 1 in PBS-T), expressed following previously published procedures [64]. A secondary goat anti-human AlexaFluor-647 antibody (100 μL, 2 μg mL 1 in PBS-T) (Thermo Fisher) was applied, incubated for 1 h in a humidified chamber and washed again as described above. Slides were dried by centrifugation after the washing step and scanned immediately using an Innopsys Innoscan 710 microarray scanner at the appropriate excitation wavelength. To ensure that all signals were in the linear range of the scanner’s detector and to avoid any saturation of the signals various gains and PMT values were employed. Images were analyzed with Mapix software (version 8.1.0 Innopsys) and processed with our home-written Excel macro. The average fluorescence intensity and SD were measured for each compound after exclusion of the highest and lowest intensities from the spot replicates (n = 4).

### Flow cytometry

The expression of NA protein on MDCK-II cells was quantified by flow cytometry using FACS analysis as previously described with few modifications [65]. Briefly, MDCK-II cells in 24-well plates were infected with MOI 1 of different viruses in three replicates. At 8 and 24 h, the supernatant (0.5mL) were firstly collected then cell sheets were dissociated by trypsin (0.250 mL) and anti-N1 polyclonal antibodies were added at a ratio 1:1000 for 1 h to ~5x10^6^ non-permeabilized single cells. After washing, secondary anti-rabbit antibodies were added 1:20000 and the cells were subjected to immuno-staining for flow cytometry analysis using a BD FACSCanto flow cytometer (BD Biosciences). The mean fluorescence intensity (MFI) of non-infected cells was subtracted from infected cells and the results were shown as ΔMFI and standard deviation of all replicates. For the detection of SA moieties, MDCK-II and MDCK-SIAT cells were infected at an MOI of 1 with rg-AL or rg-HL-16. After 2 hour incubation at 37°C cells were detached with trypsin and washed once with staining buffer. Fluorescein-labelled *Sambucus nigra* lectin (SNA; Vector Laboratories) and biotin-labelled *Maackia amurensis* lectin II (MAL II; Vector Laboratories) were used for the detection of (α-2,6) or (α-2,3) linked sialic acids, respectively, in infected and non-infected cells. After washing, MAL II binding was detected using PE-Cy7-labelled streptavidin (Biolegend). All incubation steps were carried out at 4°C in the dark. Stained infected and non-infected cells were treated with fixation buffer according to the manufacturer’s protocol (Biolegend). Reading was done using a BD FACSCanto flow cytometer (BD Biosciences). The results are the average and standard deviation of a triplicate experiment.

### Western blot

MDCK-II cells were infected with indicated viruses at an MOI of 1 and cells were collected 24 hpi and subjected for standard western blot procedures. Virus purification from allantoic fluid of eggs inoculated by rg-AL or rg-HL-16 in three independent replicates, each 20 to 30 eggs, was conducted following the published protocol [66]. NA was detected using rabbit anti-N1 antibodies or NA-universal HCA-2 antibody [67]. GAPDH, β-actin, M1 and NP proteins were detected using commercial monoclonal antibodies and peroxidase-conjugated rabbit or mouse IgG (Sigma Aldrich). Antibody binding was detected by luminescence (Supersignal West Pico chemiluminescent substrate kit, Pierce, ThermoScientific, Rockford, IL, USA) in a BioRad Versa Doc System and Quantity One software. Relative quantification of different proteins to GAPDH or β-actin of four independent experiments was determined by image J software.

### Virus purification and transmission electron microscopy

Indicated viruses were propagated in ECE in three independent experiments, 40 eggs each. Allantoic fluids were harvested and clarified through 30% sucrose in NTC buffer (100mM NaCl, 10mM Tris-HCl, 5 mM CaCl_2_) at 10000 rpm for 2 h at 4°C a Beckman SW28 rotor. Pellets were resuspended in NTE-buffer and centrifuged through a 30–60% continuous sucrose gradient in NTC as previously described [66]. Purified virions in each round were analysed by electron microscopy as previously published [68]. The samples were transferred to formvar coated TEM grids (400 mesh, Plano GmbH, Germany), stained with 1% phosphotungstic acid at pH 6.0 and analysed with a Tecnai-Spirit (FEI, Eindhoven, Germany) transmission electron microscope at an accelerating voltage of 80 kV.

### Mass spectrometry

The protein contents of three independent purified virus preparations were determined using the bicinchoninic acid assay as described by Stoscheck [69]. Between 20 and 40 μg of protein were diluted with one volume of lysis buffer (100mM Tris HCl pH 8.0, 2% SDS, 100mM DTT), sonicated, and boiled (5 min, 95°C). Samples were transferred to 30kDa cut-off ultrafiltration devices (Vivacon 500, Sartorius VN01H22) and concentrated to the dead-stop volume. Proteins were then processed with the FASP [70] with minor modifications. Peptides were desalted by solid phase extraction (Pierce C18 tips, Thermo 87784), dried by vacuum centrifugation and resuspended in 0.1% formic acid (FA). Peptides (200ng per sample) were analysed on a timsTOF Pro platform coupled to a nanoElute HPLC (Bruker, Germany) equipped with a Bruker Ten column (Bruker). Data were acquired using the PASEF mode with parameters suggested by the manufacturer of the instrument. For the chromatography, a gradient of solvent B (0.1% FA in acetonitrile) was applied from 2% to 20% (46 min) and 32% (60 min). Solvent A was 0.1% FA in MS-grade water. Protein identification and quantitation were carried out with Maxquant software (version 2.0.3.0) [71]. The sequence database was compiled from the *Gallus gallus* proteome (UP000000539), downloaded from Uniprot [72] in August 2022, and the virus-specific sequences. Carbamidomethylation was set as a fixed modification of C residues, acetylation was allowed for protein N-termini as well as the oxidation of M residues and the phosphorylation of S, T, and Y residues. Intensity-based absolute quantification (iBAQ) [73] values from Maxquant were normalized to the iBAQ of the NP as a virus-specific internal standard [74]. The relative amounts of NP-normalized and M1-normalized viral proteins were used to calculate the rg-HL-16:rg-AL ratios.

### Detection of vRNA, cRNA and mRNA

Strand-specific amplification of vRNA, cRNA and mRNA of NA was done as published [22] after infection of MDCK-II cells with an MOI of 1. The RT-qPCR was done using Power SYBR Green Kit (Thermo Fisher) and strand-specific primers. Results are the mean and standard deviation of two triplicate experiments.

### Indirect immunofluorescence and confocal laser-scanning microscopy

For immunofluorescence analysis, cells infected with an MOI of 1 of indicated viruses for 8 and 24h were washed with PBS, fixed using 4% paraformaldehyde (PFA), and blocked with 0.025% skimmed milk or 10% normal donkey serum in 0.1% Tween-20 in PBS. Rabbit anti-influenza A virus NA N1 (1:1,000), mouse anti-β-tubulin (1:100, clone: TUBB2.1; Sigma-Aldrich), rabbit anti-ZO-1 (1:200; proteintech, USA), and mouse anti-IAV NP HB-65 (1:5; ATCC) [75] were used as primary antibodies. Alexa Fluor-conjugated antibodies (1:1,000; Invitrogen, USA) against mouse IgG1, mouse IgG2a, and rabbit IgG were used as secondary antibodies. Nuclei were counterstained with Hoechst33342 (Invitrogen). Confocal images were acquired with a Leica DMI6000 TCS SP5 confocal laser-scanning microscope (Leica Microsystems, Germany) equipped with a 63x/1.40 oil immersion HCX PL APO objective and a 40x/1.10 water immersion HC PL APO objective. Image stacks were acquired with a pinhole diameter of 1 Airy unit and a z-step size of 0.35 μm and 0.5 μm, respectively. Post-processing, visualization, and analysis were done with Fiji [76] and arivis vision4D (v3.4.0).

### Animal experiment

#### Chickens

4-to-6-week-old chickens were allocated into 8 groups and inoculated intranasal with 10^4^ PFU/bird. Sentinel chickens were added 1 dpi to assess virus transmission. Birds were observed daily for 10 days for health disorders and clinical scoring and MTD were calculated as previously described [63].

#### Mice

Five-week-old BALB/c mice were allocated to 7 groups (n = 8 mice per group) in Isocages. Six groups were IN-inoculated with 10^5^ PFU/mouse of the indicated viruses and one control group was IN-inoculated with 50 μl PBS after inhalation anesthesia using isoflurane. BW of each mouse was measured daily and mice having lost >25% of their body or exhibited severe neurological signs were euthanized using isoflurane and decapitation. Clinical signs, BW, survival rate and MTD were recorded. Infectious virus titers in the lungs and brain homogenates of three euthanized mice per group were determined by plaque assay. Immune response was studied in BALB/C mice (n = 3). For immune response, spleens were collected and single cell suspension was subjected for flow cytometry analysis using legends against murine surface antigens (Biolegend) of CD4, CD8, and CD69 and analyzed by FACS machine (BD Biosciences).

#### Ferrets

Six-to-nine month-old influenza-free ferrets were housed in cages three days before the infection for acclimatization. Ferrets were obtained from the quarantine stables at the FLI, where they were reared and kept. At day 0, two ferrets were IN-inoculated with 5x10^5^ PFU/mL of indicated viruses. One ferret was inoculated with PBS as sham control. All the infection was done after deep anesthesia using Isoflurane. Two sentinel ferrets were added 1 dpi to the infected ones in two cages. Each cage contained one inoculated and one sentinel animal. Nasal wash, 1 mL PBS, under isoflurane sedation was collected before inoculation and at 2, 4, 7 and 10 dpi using pipette. Clinical examination was done daily. A loss of 25% of body weight or severe neurological signs were the humane endpoint for euthanization. Nasal washes at indicated time points were tested for viral RNA using quantitative RT-qPCR as previously described [77] after RNA extraction using NucleoSpin 8/96 PCR Clean-up Core Kit (Macherey & Nagel) according to the manufacturer instructions. The PCR was conducted in AriaMx Real-Time PCR system (Agilent Technologies). Anti-NP antibodies were determined using ID screen Influenza A Antibody Competition Multispecies ELISA kit (IDvet) and plates were read in a Tecan ELISA reader.

#### Genetic analysis and structural modelling

Amplification of all gene segments and Sanger sequencing of the rg-HL-16 and recombinant viruses was conducted as previously done [3, 78]. All available NA genes of Egyptian A/H5N1 from 16-02-2006 to 31-12-2016 and Asian viruses from 1997 to 2016 were retrieved from the GenBank and GISAID. Sequences were aligned and edited using Geneious. Mutations in the NA compared to the parent 2.2.1 virus (A/chicken/Egypt/NLQP-06207/2006) were analyzed. 3D structures were generated by SWISS MODEL (http://swissmodel.expasy.org/). The 2D structure of N-acetylneuraminic acid [Nue5Ac], the most abundant natural ligand for binding to NA was compiled using ChemDraw, 3D structure of Nue5Ac ligand was constructed using Chem 3D ultra 15.0 software Molecular Modeling and Analysis; Cambridge Soft Corporation, USA [2014], then they were energetically minimized by using MOPAC [semi-empirical quantum mechanics], and saved as MDL MolFile [*.mol]. The 3D protein structures, created by homology modeling using SWISS-MODEL server in PDB format were loaded on to Molegro Virtual Docker (MVD) 6.0 (2013) platform for docking process. Potential binding sites have been identified using the built-in cavity detection algorithm of MVD. Subsequently, the docking process between Nue5Ac and the active sites of the different NA strains were carried out using MolDock SE as the scoring function of MVD [79]. The best conformations for each docking process were selected based on the lowest docked binding energy (MolDock score).

#### Statistical analysis

All statistical analyses were performed using GraphPad version 8.0 and the data sets were checked for normal distribution via Q-Q-Plots. Results of relative NA-expression in purified virions were analyzed using unpaired t-tests with Welchs correction. Ct-values for different RNA strands, plaque size, receptor-binding, receptor distribution in MDCK cells, replication kinetics, ELLA-Assay, virus accumulation, supernatant-titers immune response, mice bodyweight values, and ferret data were analyzed using one-way ANOVA tests, while the NA activity against MUNANA were analyzed with two-way ANOVA. In addition, the 50%-NA-activity against MUNANA was calculated for each virus compared to the results obtained to those of rg-AL by plotting the three independent replicates separately and analysis using unpaired t-test and Welchs-correction (n = 3). Kruskal-Wallis test was used for virus isolation in mice organs and Log-Rank test was used for the survival curves. Results are shown as mean and standard deviation and were statistically significant at p value < 0.05. The graphical abstract was generated by BioRender.

#### Biosafety

Experiments with highly pathogenic viruses in this study were conducted in the biosafety level 3 (BSL3) laboratory and stable facilities at the FLI. The bio-risk officer and committee for dual-use research of concern were consulted and agreed on the animal experiments. Replication of the indicated viruses in NHBE cells was done at the BSL3 facilities of the Institute of Medical Virology, Justus Liebig University Giessen, Germany. The experiments in this study were done, documented and closely supervised by experienced researchers.

### Conflicts of interest

The authors declare no conflict of interest. The founding sponsors had no role in the design of the study; in the collection, analyses, or interpretation of data; in the writing of the manuscript, and in the decision to publish the results.

## Supporting information

S1 Fig Molecular docking of NA from different viruses using Neu5Ac sialic acid legend. Molecular docking of NA of avian-like (rg-AL), AL with L204M (rg-AL-204M) and with four mutations (rg-HL-08) or indicated HxNx viruses with Neu5Ac sialic acid ligand using Moldock. In parenthesis is the Moldock score, which estimates the binding interaction (Gibbs free energy) between the target receptor and the ligand in kcal/mol. Predicted interaction sites in the NA with SA legend are listed, which indicate potential conformational changes in the sialidase pocket after insertion of the indicated mutations in the NA. Red arrows indicate new potential interaction sites were predicted and triangles indicate abolishing interaction sites after L204M mutation.
(TIF)

S2 Fig Replication kinetics of H5N1 viruses in mammalian cells. Replication kinetics of H5N1 viruses in Madin-Darby canine kidney cells type II (MDCK-II) (A) and human lung adenocarcinoma cell line A549 (B). Cells were infected with an MOI of 0.001 for indicated time points in three independent replicates. Results are shown as mean and standard deviation of plaque forming units/ml (PFU/ml). Dashed lines indicate the predicted detection limit of plaque assay in this study. Asterisks indicate significant differences at p < 0.05 for each virus in comparison with rg-AL.
(TIF)

S3 Fig Relative NA expression in different cells. Detection of NA of indicated viruses with or without mutation in position 204 after infection of MDCK-II cells at an MOI of 1 for 24 h. NA was detected using universal anti-NA-HCA-2 antibody (A, B). NA expression in chicken fibroblast DF1 (C, D) or A549 (E, F) cells transfected for 24 h with 1μg of the expression vector pCAGGS containing the complete NA sequence from rg-AL, rg-AL-204M or rg-HL-16 (C-F). Detection of NA and M1 expression in MDCK-II cells infected with an MOI of 1 with the indicated H7N7/H5N1 viruses for 24 h (G-H). NA was detected by rabbit polyclonal anti-N1-antibodies as primary antibody in standard Western Blot. The chemiluminescence signal of NA was divided by the value for M1 and normalized to β-actin (A, B, G, H) or the value for β-actin (C-F). The expression of NA was calculated as mean and standard deviation of three independent experiments. Asterisks indicate significant differences at p < 0.05 (B, D, F, H).
(TIF)

S4 Fig Selection of NA of avian or human-like H5N1. NA selection was studied after co-transfection of HEK293T cells with 9 plasmids (8 full set of rg-HL-16 and NA of rg-AL) at NA ratio of 1:1 (1 μg each) in two different rounds (left and middle sides) or at ratio 10:1 (1 μg NA of rg-HL-16 and 0.1 μg NA of rg-AL) (right side). Plaques were selected and subjected for Sanger sequencing. The number of plaques positive for specified NA variant is shown. Black colour refers to avian NA, blue for human-like NA and grey for plaques mixed with mixed NAs.
(TIF)

S5 Fig Removal of avian and human-like sialic acid receptors during virus entry. MDCK-SIAT and MDCK-II cells, which are rich in 2,6-SA and 2,3-SA, respectively, were infected with rg-HL-16 and rg-AL at MOI of 1 at 37°C for two hours only. The amount of 2,6-SA and 2,3-SA was quantified using flow cytometry to measure signals of fluorescein-labelled *Sambucus nigra* lectin and biotin-labelled *Maackia amurensis* lectin II compared to naïve uninfected cells (NC). Reading was done using a BD FACSCanto flow cytometer (BD Biosciences). The results are the average and standard deviation of a triplicate experiment. Asterisks indicate significant differences at p < 0.05.
(TIF)

S6 Fig Replication of H5N1 viruses in ferret primary airway epithelial cells. Airway epithelial cells obtained from ferrets were fully differentiated into a polarized, pseudostratified, and multicellular respiratory epithelium under ALI conditions. Depicted are a top-down view (left; green: β-tubulin, grayscale: ZO-1, blue: nuclei) and a cross section (right; green: KRT5, red: Muc5AC, blue: nuclei) of the cultured cells. Scale bar: 40 μm (A). In comparison to H3N2, NA variants of H5N1 exhibited a reduced specific infectivity on fully-differentiated FAECs. Each infection (5 × 10^5^ PFU/insert) was performed in duplicate on fully-differentiated FAECs from an independent donor ferret (total of three donor ferrets; n = 6 per virus). Representative images are shown for each condition. Scale bar: 50 μm (overview), 15 μm (detail) (B).
(PNG)

S7 Fig The impact of NA mutations on early cell-mediated immune response in mice. Early activation of CD4+ or CD8+ T cells in the spleen of mice at 3 dpi expressed as mean and standard deviation. Spleens were collected and single cell suspension was subjected for flow cytometry analysis using legends against murine surface antigens of CD4, CD8, and CD69. Asterisks indicate significant differences compared to rg-AL at p < 0.05.
(TIF)

S8 Fig The impact of NA mutations on the virulence, replication and transmission of H5N1 viruses in ferrets. Ferrets (N = 2 per group) were inoculated intranasally with 5 x 10^5^ PFU of indicated viruses in 0.1 mL and the sham group received 100μL PBS. Inoculation was done after isoflurane anaesthesia. Two sentinels were added 1 dpi. Pathogenicity index was calculated as the sum of daily clinical scoring values (S3 Table) during 10-day observation period and shown in the y-axis. Clinical scoring: 0 = normal (i.e. playful and alerted), 1 = mild illness: one clinical sign (inappetance, closed eyes or reduced movement but still alert), 2 = moderate illness: two signs (and not playful), 3 = severe flu-like illness exhibiting all these signs together: nasal discharge, dyspnoea, sneezing, lethargy, shivering and eye closed (A). Viral RNA detection in nasal washes (B,C) for 10 dpi of virus-inoculated and cohoused ferrets. Inoculated ferrets (n = 2) are shown in red and contact ferrets (n = 2) are shown in blue. Ferrets indicated by dashed or continuous lines were co-housed together. Virus excretion was determined by quantitative real-time RT-PCR in nasal washes using standard curves targeting the M gene of different dilutions of rg-HL-16. Results are shown as individual data and mean of positive samples. Asterisks indicate significant differences at p < 0.05.
(TIF)

S1 Table List of viruses used in this study. JLU = Justus Liebig Universität, Giessen, Germany, FLI = Friedrich-Loeffler-Institut, Germany, UC-Davis = University of California, USA, IZSVe = Istituto Zooprofilattico Sperimentale delle Venezie, Italy. *Only the NA was cloned from the indicated HPAIV H5N5/H5N6/H5N8 viruses. Other gene segments were from PR8. ** GenBank accession numbers, while unmarked accession numbers refer to GISAID numbers. Only H5N1 and H5N1/R65 are highly pathogenic in chickens, and other viruses are low pathogenic.
(DOCX)

S2 Table Identity matrices and amino acid differences between human H5N1 and H7N7 used in this study. Residues numbers from the first Methionine in the open reading frame and expressed in the order of aa in H5N1, residue number, aa in H7N7, Δ = deletion in the NS1 linker domain.
(DOCX)

S3 Table Clinical scoring and seroconversion for direct inoculated and co-housed ferrets. Ferrets were inoculated intranasally with 5 x 10^5^ PFU of indicated viruses in 0.1 mL and the sham group received 100μL PBS. Inoculation was done after isoflurane anaesthesia. Sentinels were added 1 dpi. Clinical scoring: empty cells = normal (i.e. playful and alerted), 1 = mild illness: one clinical sign (inappetence, closed eyes or reduced movement but still alert), 2 = moderate illness: two signs (and not playful), 3 = severe illness: nasal discharge, dyspnoea, sneezing, lethargy, shivering and eye closed. Serum samples collected after euthanization 10 dpi were tested after heat inactivation by ELISA. Each sample was tested twice.
(DOCX)
