# Supplementary figures and images for: Phenotypic effects of mutations observed in the neuraminidase of human origin H5N1 influenza A viruses
Source: PLoS Pathog. 2023 Feb 6;19(2):e1011135. doi: 10.1371/journal.ppat.1011135 (PMC9934401; doi:10.1371/journal.ppat.1011135)

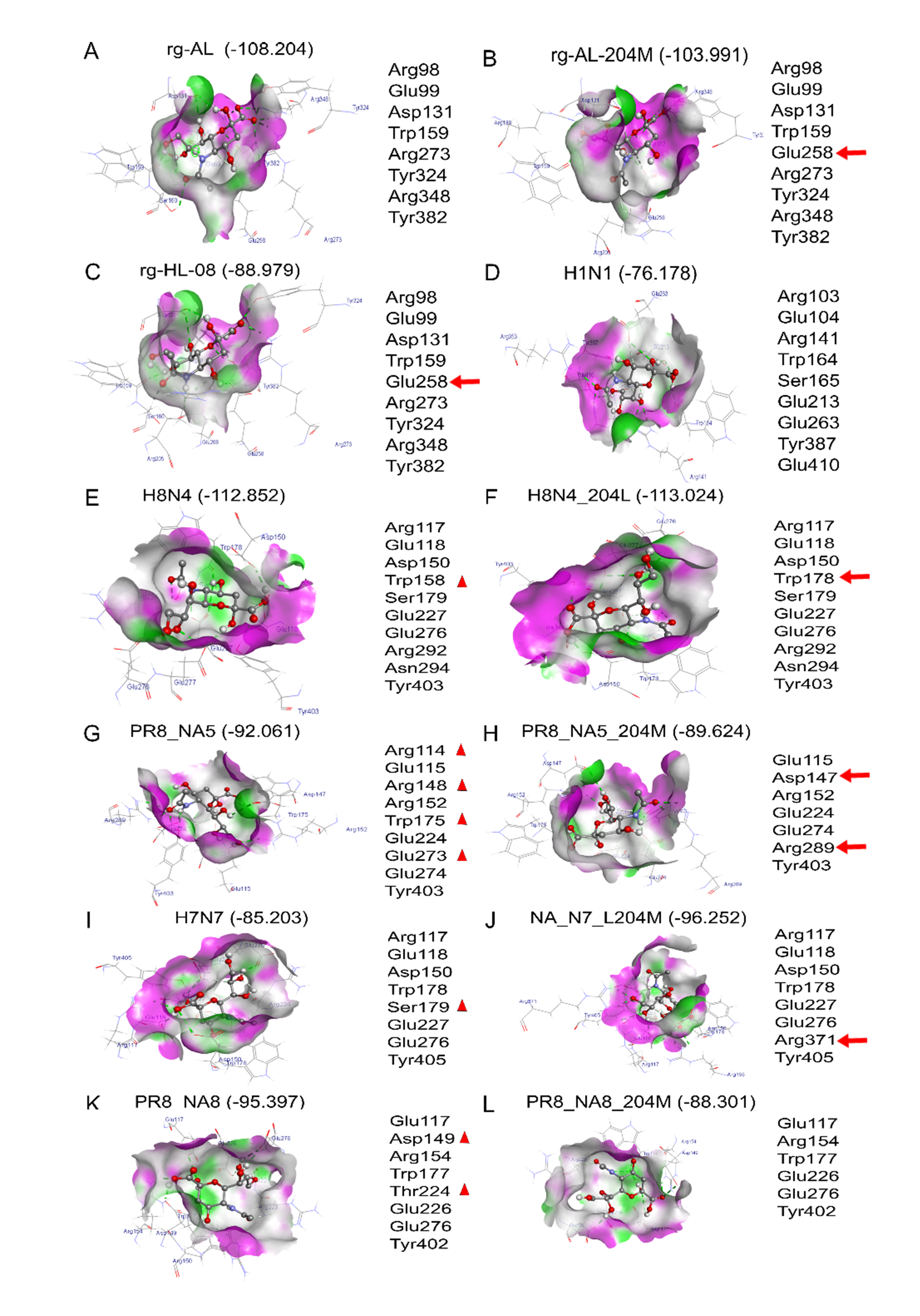

Supplement: S1 Fig — Molecular docking of NA of avian-like (rg-AL), AL with L204M (rg-AL-204M) and with four mutations (rg-HL-08) or indicated HxNx viruses with Neu5Ac sialic acid ligand using Moldock. In parenthesis is the Moldock score, which estimates the binding interaction (Gibbs free energy) between the target receptor and the ligand in kcal/mol. Predicted interaction sites in the NA with SA legend are listed, which indicate potential conformational changes in the sialidase pocket after insertion of the indicated mutations in the NA. Red arrows indicate new potential interaction sites were predicted and triangles indicate abolishing interaction sites after L204M mutation. (TIF) [file ppat.1011135.s001.tif]

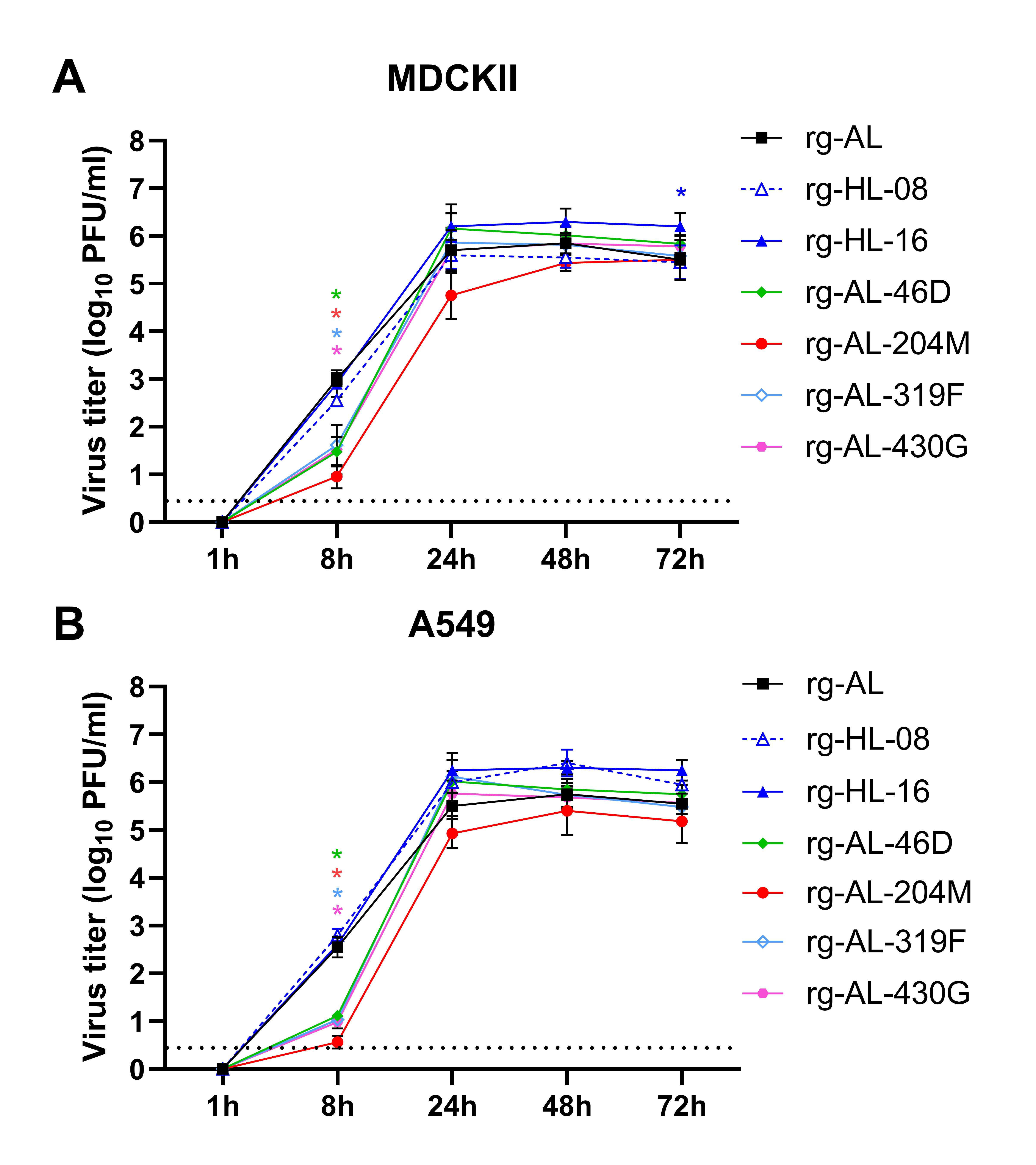

Supplement: S2 Fig — Replication kinetics of H5N1 viruses in Madin-Darby canine kidney cells type II (MDCK-II) (A) and human lung adenocarcinoma cell line A549 (B). Cells were infected with an MOI of 0.001 for indicated time points in three independent replicates. Results are shown as mean and standard deviation of plaque forming units/ml (PFU/ml). Dashed lines indicate the predicted detection limit of plaque assay in this study. Asterisks indicate significant differences at p < 0.05 for each virus in comparison with rg-AL. (TIF) [file ppat.1011135.s002.tif]

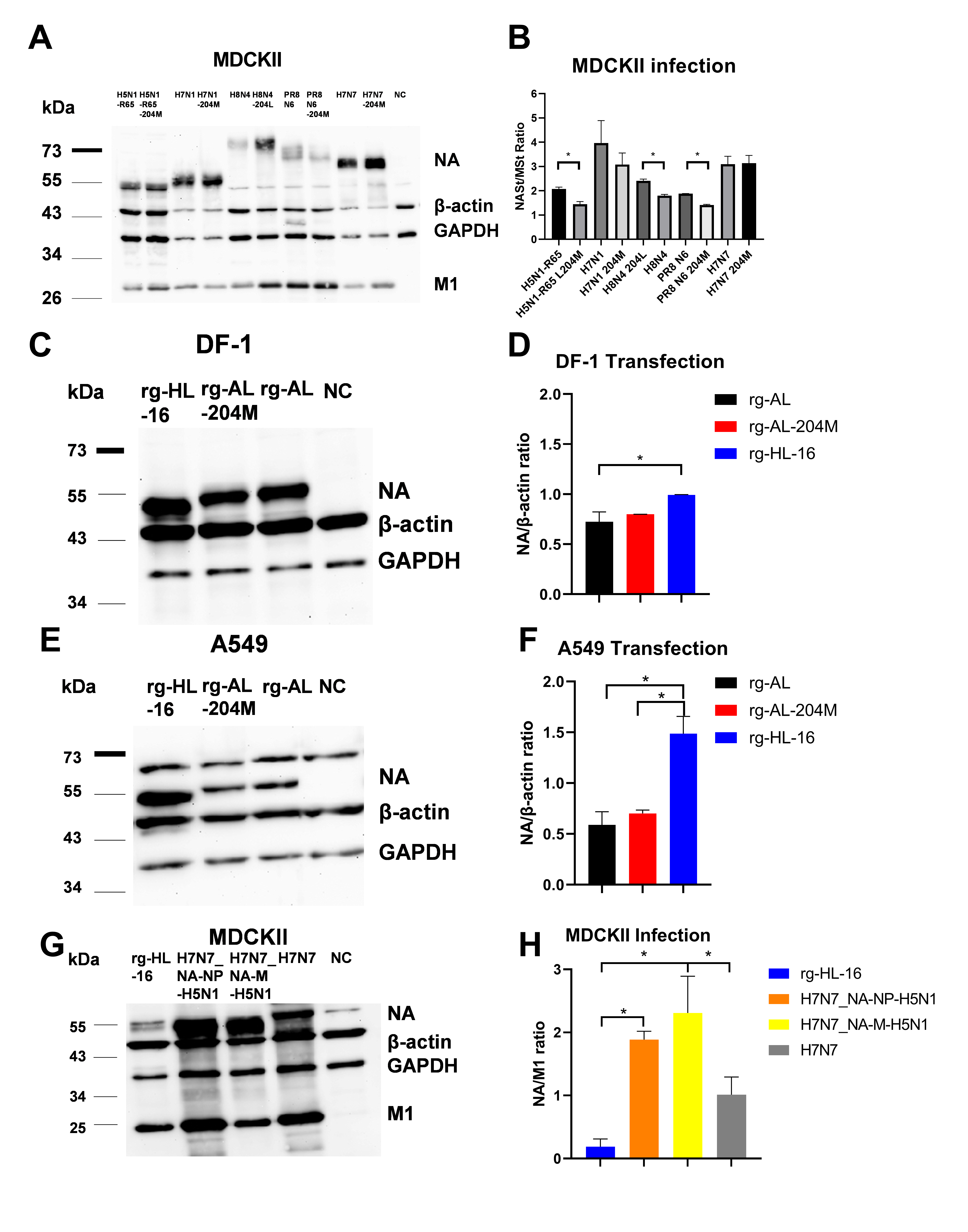

Supplement: S3 Fig — Detection of NA of indicated viruses with or without mutation in position 204 after infection of MDCK-II cells at an MOI of 1 for 24 h. NA was detected using universal anti-NA-HCA-2 antibody (A, B). NA expression in chicken fibroblast DF1 (C, D) or A549 (E, F) cells transfected for 24 h with 1μg of the expression vector pCAGGS containing the complete NA sequence from rg-AL, rg-AL-204M or rg-HL-16 (C-F). Detection of NA and M1 expression in MDCK-II cells infected with an MOI of 1 with the indicated H7N7/H5N1 viruses for 24 h (G-H). NA was detected by rabbit polyclonal anti-N1-antibodies as primary antibody in standard Western Blot. The chemiluminescence signal of NA was divided by the value for M1 and normalized to β-actin (A, B, G, H) or the value for β-actin (C-F). The expression of NA was calculated as mean and standard deviation of three independent experiments. Asterisks indicate significant differences at p < 0.05 (B, D, F, H). (TIF) [file ppat.1011135.s003.tif]

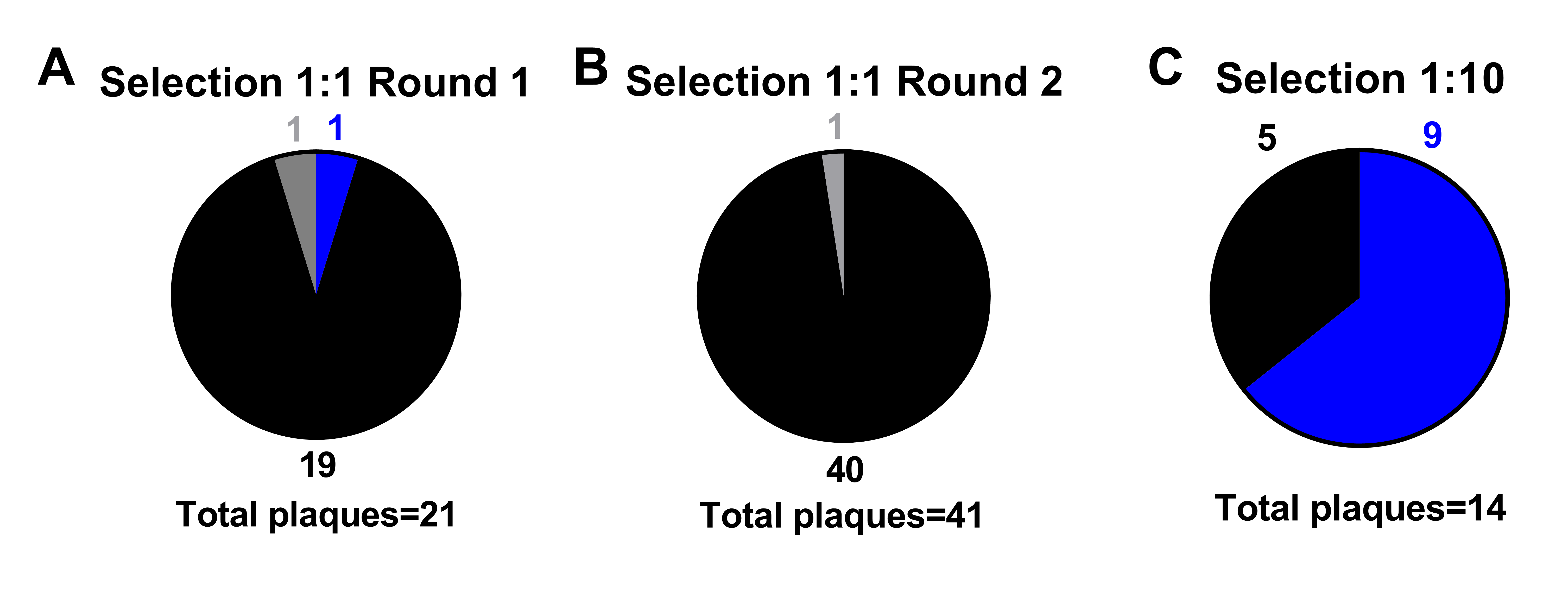

Supplement: S4 Fig — NA selection was studied after co-transfection of HEK293T cells with 9 plasmids (8 full set of rg-HL-16 and NA of rg-AL) at NA ratio of 1:1 (1 μg each) in two different rounds (left and middle sides) or at ratio 10:1 (1 μg NA of rg-HL-16 and 0.1 μg NA of rg-AL) (right side). Plaques were selected and subjected for Sanger sequencing. The number of plaques positive for specified NA variant is shown. Black colour refers to avian NA, blue for human-like NA and grey for plaques mixed with mixed NAs. (TIF) [file ppat.1011135.s004.tif]

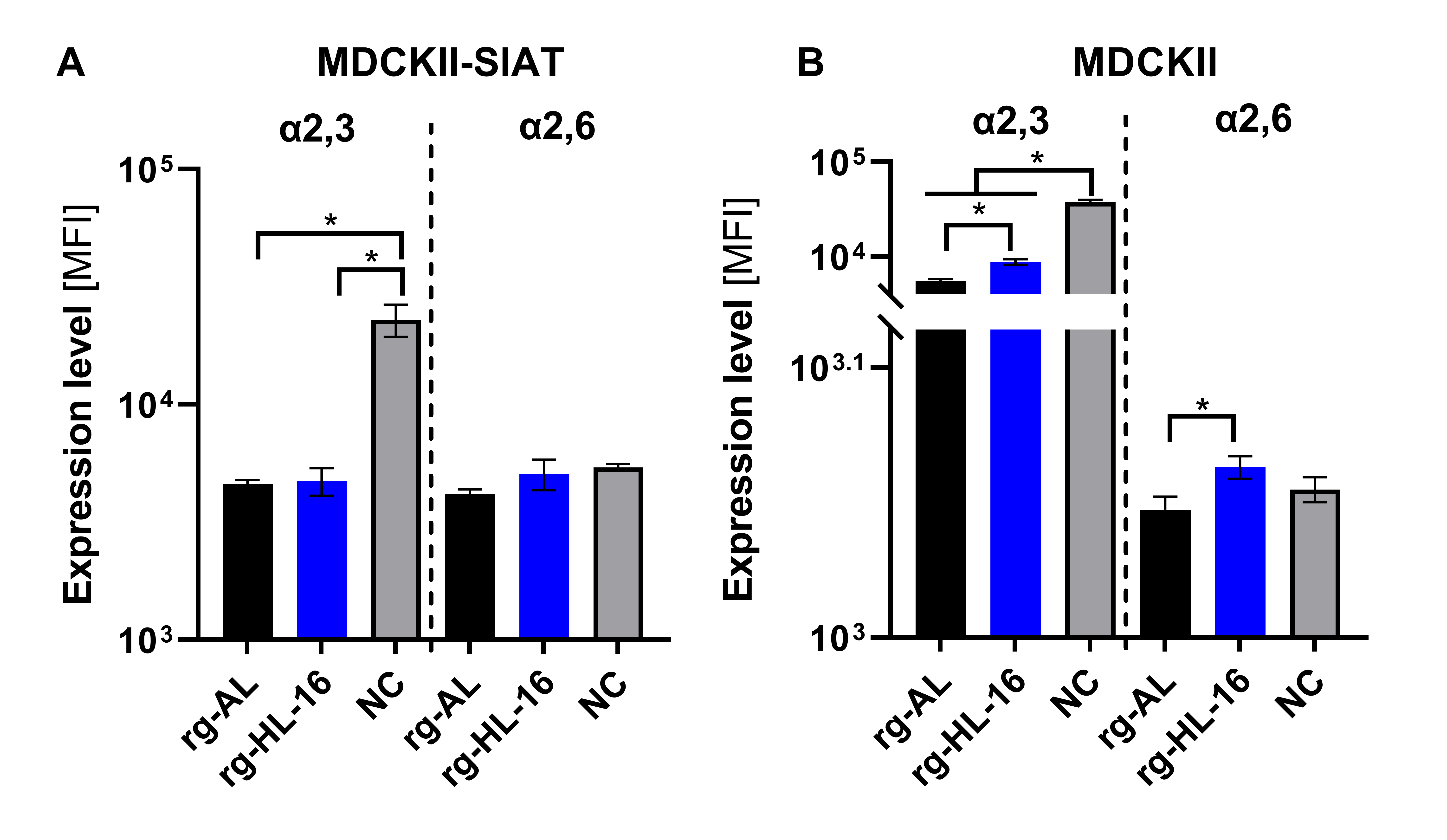

Supplement: S5 Fig — MDCK-SIAT and MDCK-II cells, which are rich in 2,6-SA and 2,3-SA, respectively, were infected with rg-HL-16 and rg-AL at MOI of 1 at 37°C for two hours only. The amount of 2,6-SA and 2,3-SA was quantified using flow cytometry to measure signals of fluorescein-labelled Sambucus nigra lectin and biotin-labelled Maackia amurensis lectin II compared to naïve uninfected cells (NC). Reading was done using a BD FACSCanto flow cytometer (BD Biosciences). The results are the average and standard deviation of a triplicate experiment. Asterisks indicate significant differences at p < 0.05. (TIF) [file ppat.1011135.s005.tif]

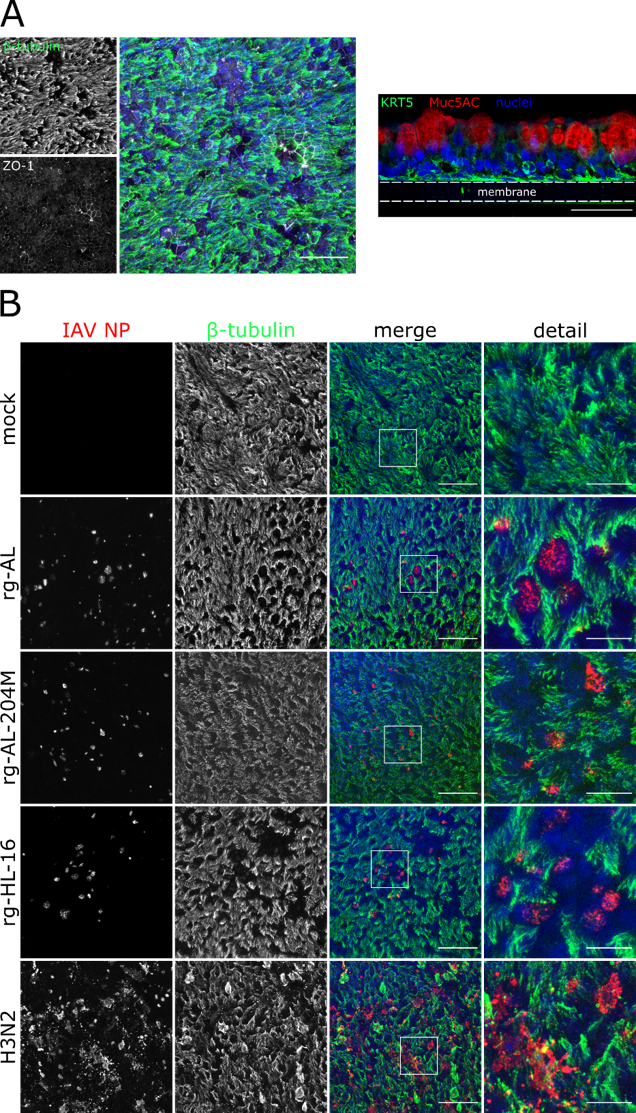

Supplement: S6 Fig — Airway epithelial cells obtained from ferrets were fully differentiated into a polarized, pseudostratified, and multicellular respiratory epithelium under ALI conditions. Depicted are a top-down view (left; green: β-tubulin, grayscale: ZO-1, blue: nuclei) and a cross section (right; green: KRT5, red: Muc5AC, blue: nuclei) of the cultured cells. Scale bar: 40 μm (A). In comparison to H3N2, NA variants of H5N1 exhibited a reduced specific infectivity on fully-differentiated FAECs. Each infection (5 × 10^5^ PFU/insert) was performed in duplicate on fully-differentiated FAECs from an independent donor ferret (total of three donor ferrets; n = 6 per virus). Representative images are shown for each condition. Scale bar: 50 μm (overview), 15 μm (detail) (B). (PNG) [file ppat.1011135.s006.png]

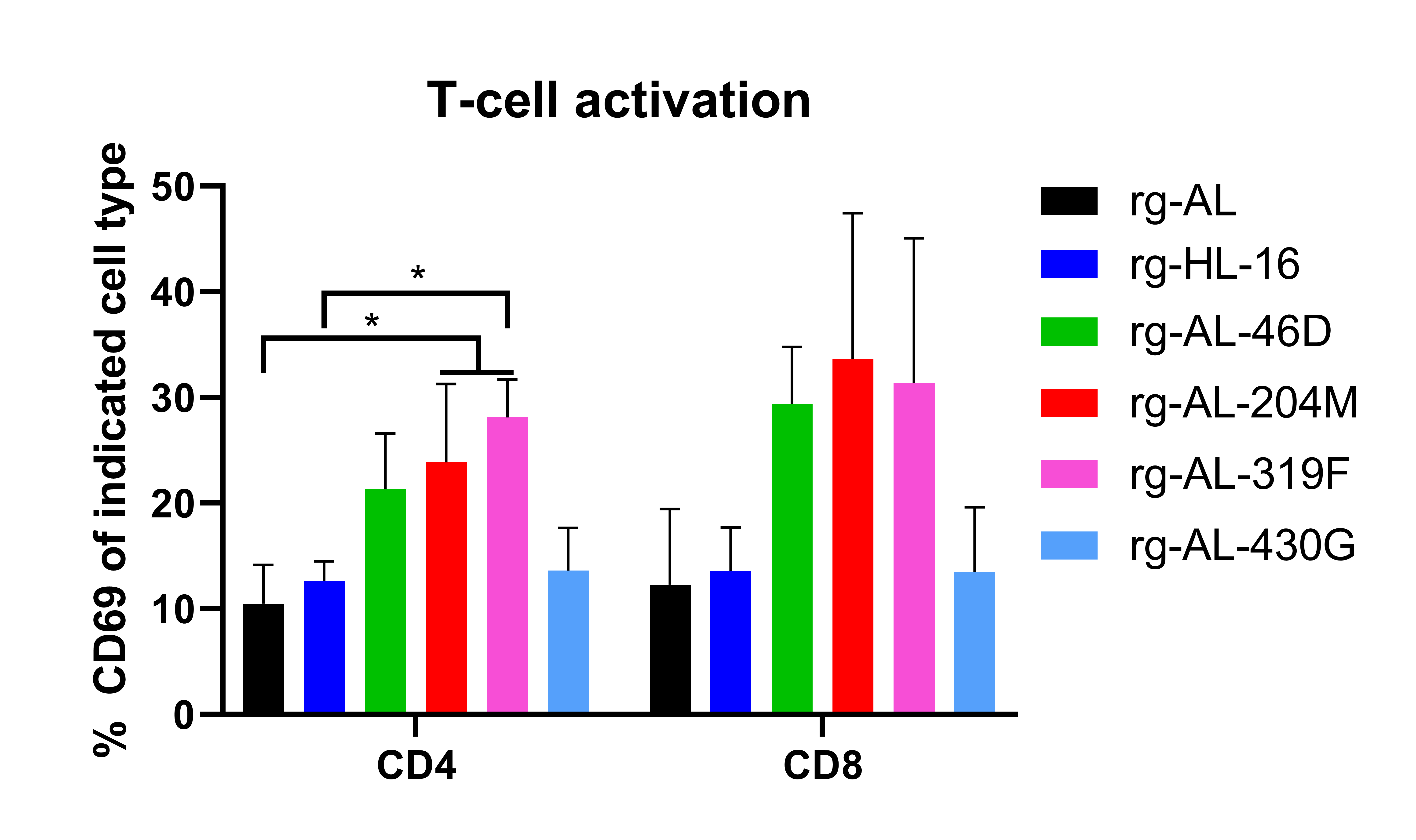

Supplement: S7 Fig — Early activation of CD4+ or CD8+ T cells in the spleen of mice at 3 dpi expressed as mean and standard deviation. Spleens were collected and single cell suspension was subjected for flow cytometry analysis using legends against murine surface antigens of CD4, CD8, and CD69. Asterisks indicate significant differences compared to rg-AL at p < 0.05. (TIF) [file ppat.1011135.s007.tif]

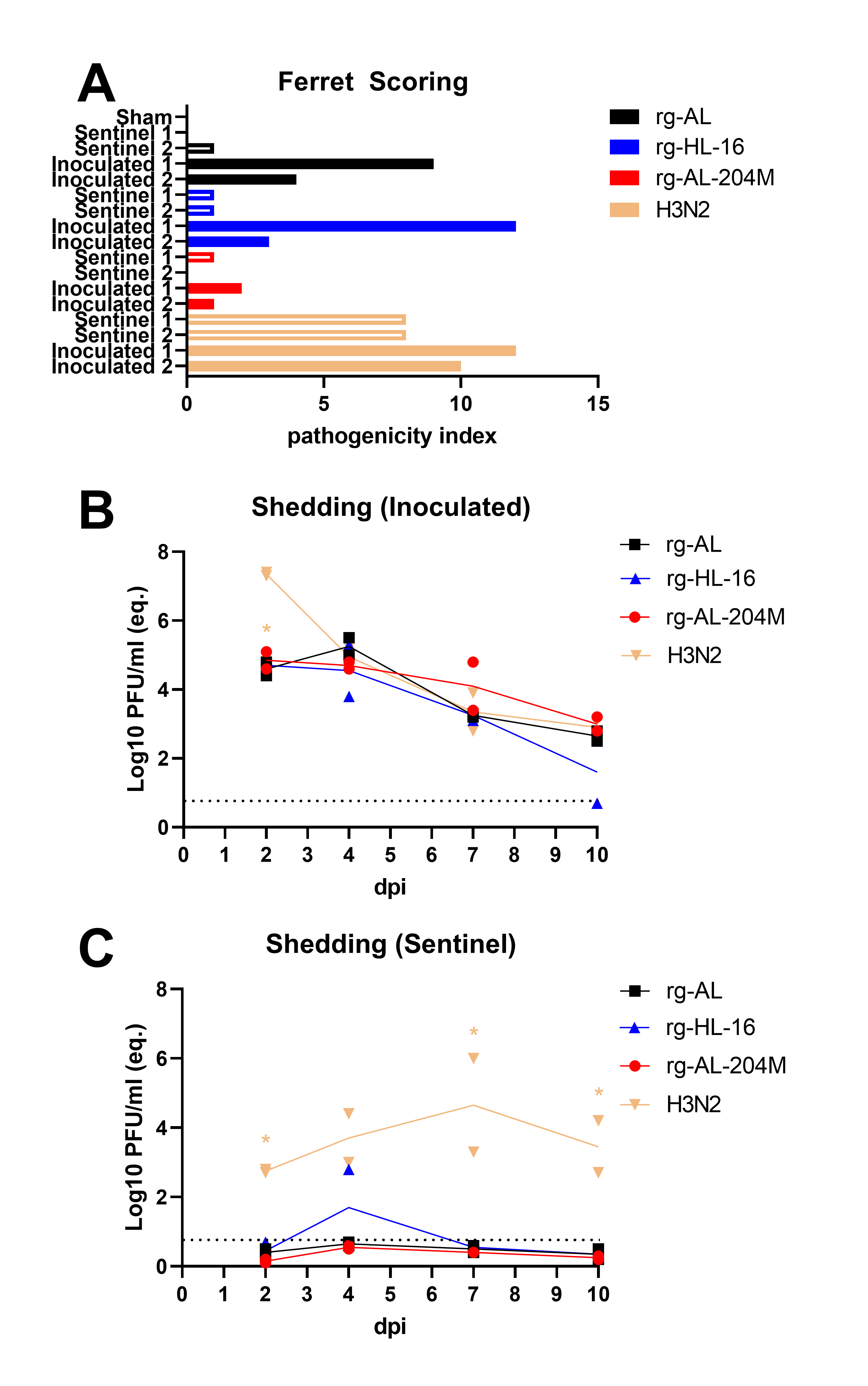

Supplement: S8 Fig — Ferrets (N = 2 per group) were inoculated intranasally with 5 x 10^5^ PFU of indicated viruses in 0.1 mL and the sham group received 100μL PBS. Inoculation was done after isoflurane anaesthesia. Two sentinels were added 1 dpi. Pathogenicity index was calculated as the sum of daily clinical scoring values (S3 Table) during 10-day observation period and shown in the y-axis. Clinical scoring: 0 = normal (i.e. playful and alerted), 1 = mild illness: one clinical sign (inappetance, closed eyes or reduced movement but still alert), 2 = moderate illness: two signs (and not playful), 3 = severe flu-like illness exhibiting all these signs together: nasal discharge, dyspnoea, sneezing, lethargy, shivering and eye closed (A). Viral RNA detection in nasal washes (B,C) for 10 dpi of virus-inoculated and cohoused ferrets. Inoculated ferrets (n = 2) are shown in red and contact ferrets (n = 2) are shown in blue. Ferrets indicated by dashed or continuous lines were co-housed together. Virus excretion was determined by quantitative real-time RT-PCR in nasal washes using standard curves targeting the M gene of different dilutions of rg-HL-16. Results are shown as individual data and mean of positive samples. Asterisks indicate significant differences at p < 0.05. (TIF) [file ppat.1011135.s008.tif]
