# Supplementary material for: Phenotypic effects of mutations observed in the neuraminidase of human origin H5N1 influenza A viruses
Source: PLoS Pathog. 2023 Feb 6;19(2):e1011135. doi: 10.1371/journal.ppat.1011135 (PMC9934401; doi:10.1371/journal.ppat.1011135)
Supplement: S1 Table — JLU = Justus Liebig Universität, Giessen, Germany, FLI = Friedrich-Loeffler-Institut, Germany, UC-Davis = University of California, USA, IZSVe = Istituto Zooprofilattico Sperimentale delle Venezie, Italy. *Only the NA was cloned from the indicated HPAIV H5N5/H5N6/H5N8 viruses. Other gene segments were from PR8. ** GenBank accession numbers, while unmarked accession numbers refer to GISAID numbers. Only H5N1 and H5N1/R65 are highly pathogenic in chickens, and other viruses are low pathogenic. (DOCX) [file ppat.1011135.s009.docx]

**Supplementary Table S1:** List of viruses used in this study

|  | Abbreviation | Nomenclature | NA Accession numbers | Source |
| --- | --- | --- | --- | --- |
| 1 | H1N1 | A/Giessen/8/2018 | EPI1944223 | JLU Giessen |
| 2 | PR8 | A/PR/8/34(H1N1) | EF190976** | J. Stech, FLI |
| 3 | H3N2 | A/Victoria/3/75 | CY113183** | JLU Gießen |
| 4 | H4N2 | A/quail/California/D113023808/2012 | AHG26587** | B. Crossley, UC-Davis |
| 5 | H5N1 | A/turkey/Egypt/AR1507/2016 | EPI827071 | Alexandria University, Egypt |
| 6 | H5N1/R65 | A/swan/Germany/R65/2006 | ABE26830 | T. Harder-FLI |
| 7 | PR8_N5 | A/turkey/Germany-SH/R425/2017(H5N5)* | EPI888420 | T. Harder-FLI |
| 8 | PR8_N6 | A/common pochard/Germany-BY/AR09-18-L02421/2017(H5N6)* | EPI1139716 | T. Harder-FLI |
| 9 | H5N8 | A/tufted duck/Germany/8444/2016 (H5N8)* | EPI1753774 | T. Harder-FLI |
| 10 | H7N1 | A/chicken/Italy/473/1999 | EPI624434 | T. Harder-FLI  (I. Capua- IZSVe) |
| 11 | H7N7 | A/chicken/Germany/R1361/2011 | EPI772737 | T. Harder-FLI |
| 12 | H8N4 | A/turkey/Ontario/6118/68 | AY207528** | T. Harder-FLI |

JLU= Justus Liebig Universität, Giessen, Germany, FLI= Friedrich-Loeffler-Institut, Germany, UC-Davis= University of California, USA, IZSVe= Istituto Zooprofilattico Sperimentale delle Venezie, Italy

*Only the NA was cloned from the indicated HPAIV H5N5/H5N6/H5N8 viruses. Other gene segments were from PR8. ** GenBank accession numbers, while unmarked accession numbers refer to GISAID numbers.

Only H5N1 and H5N1/R65 are highly pathogenic in chickens, and other AIV are low pathogenic.
