# Supplementary material for: Phenotypic effects of mutations observed in the neuraminidase of human origin H5N1 influenza A viruses
Source: PLoS Pathog. 2023 Feb 6;19(2):e1011135. doi: 10.1371/journal.ppat.1011135 (PMC9934401; doi:10.1371/journal.ppat.1011135)
Supplement: S2 Table — Residues numbers from the first Methionine in the open reading frame and expressed in the order of aa in H5N1, residue number, aa in H7N7, Δ = deletion in the NS1 linker domain. (DOCX) [file ppat.1011135.s010.docx]

**Supplementary Table S2:** Identity matrices and amino acid differences between human H5N1 and H7N7 used in this study

| Segment | Nucleotide | | Amino acid | | |
| --- | --- | --- | --- | --- | --- |
|  | Identity (%) | No. of differences | Identity (%) | No. of differences | Differences* |
| PB2 | 86.2 | 319 | 96.4 | 27 | M28V, I64M, I66M, R80K, T105K, A106T, N129T, T147I, L183S, R197K, T224I, K249E, M292V, I315M, T339K, K340R, Q368R, K369R, N390D, T451I, V529I, V545I, I570M, K627E, I649V, T661A, N740D |
| PB1 | 89.0 | 225 | 98.0 | 15 | V14A, K57T, G104E, I113V, I149V, E172D, I182T, R214K, K215R, R353K, K386R, R387K, K577R, N694S, P756Q |
| PA | 90.8 | 198 | 96.1 | 28 | S58G, I62V, T85M, V94I, T129I, A183T, K204R, K269R, S287A, G321T, T337A, M342L, V348I, T359N, R367K, R388S, N394D, T400S, E448A, L482M, V554I, R615K, T618K, R626K, S653P, L707F, A712T, N716K |
| NP | 91.7 | 126 | 97.2 | 14 | S34G, R77K, K98R, V105M, V109I, S129A, T350A, T371M, A373T, N377S, I408V, V477T, K452R, N482S |
| M1 | 91.8 | 62 | 96.8 | 8 | K27R, M59I, K95R, K101R, A166V, N224S, R230K, N232D |
| M2 | 95.3 | 15 | 95.3 | 5 | E23G, R27K, F59C, M74T, N91S |
| NS1 | 90.9 | 63 | 90.4 | 22 | K44R, K55E, K71E, TIASV81-85Δ, T112A, K118R, T127N, Y138F, T155A, S171D, I187T, V188L, I206S, D207N, L212P, S216P, D217K, A225T |
| NS2 | 93.6 | 25 | 94.7 | 7 | V24M, A32G, R44Q, L59V, F65L, I70S, I93V |

* Residues numbers from the first Methionine in the open reading frame and expressed in the order of aa in H5N1, residue number, aa in H7N7, Δ= deletion in the NS1 linker domain
