# Supplementary material for: Phenotypic effects of mutations observed in the neuraminidase of human origin H5N1 influenza A viruses
Source: PLoS Pathog. 2023 Feb 6;19(2):e1011135. doi: 10.1371/journal.ppat.1011135 (PMC9934401; doi:10.1371/journal.ppat.1011135)
Supplement: S3 Table — Ferrets were inoculated intranasally with 5 x 10^5^ PFU of indicated viruses in 0.1 mL and the sham group received 100μL PBS. Inoculation was done after isoflurane anaesthesia. Sentinels were added 1 dpi. Clinical scoring: empty cells = normal (i.e. playful and alerted), 1 = mild illness: one clinical sign (inappetence, closed eyes or reduced movement but still alert), 2 = moderate illness: two signs (and not playful), 3 = severe illness: nasal discharge, dyspnoea, sneezing, lethargy, shivering and eye closed. Serum samples collected after euthanization 10 dpi were tested after heat inactivation by ELISA. Each sample was tested twice. (DOCX) [file ppat.1011135.s011.docx]

**Supplementary Table S3:** Clinical scoring and seroconversion for direct inoculated and co-housed ferrets

| Group | Ferret | Day post inoculation | | | | | | | | | | | | Serology |
| --- | --- | --- | --- | --- | --- | --- | --- | --- | --- | --- | --- | --- | --- | --- |
|  |  | 0 | 1 | 2 | 3 | 4 | 5 | 6 | 7 | 8 | 9 | 9 | 10 |  |
| Sham | (PBS |  |  |  |  |  |  |  |  |  |  |  |  | negative |
| rg-AL-204M | Sentinel 1 |  |  | 1 |  |  |  |  |  |  |  |  |  | negative |
|  | Sentinel 2 |  |  |  |  |  |  |  |  |  |  |  |  | negative |
|  | Inoculated 1 |  |  | 1 | 1 |  |  |  |  |  |  |  |  | **positive** |
|  | Inoculated 2 |  |  | 1 |  |  |  |  |  |  |  |  |  | **positive** |
| rg-AL | Sentinel 1 |  |  |  |  |  |  |  |  |  |  |  |  | negative |
|  | Sentinel 2 |  |  | 1 |  |  |  |  |  |  |  |  |  | negative |
|  | Inoculated 1 |  |  | 1 | 2 | 3 | 1 | 1 | 1 |  |  |  |  | **positive** |
|  | Inoculated 2 |  |  |  | 1 | 2 | 1 |  |  |  |  |  |  | **positive** |
| rg-HL-16 | Sentinel 1 |  |  | 1 |  |  |  |  |  |  |  |  |  | negative |
|  | Sentinel 2 |  |  |  |  | 1 |  |  |  |  |  |  |  | **positive** |
|  | Inoculated 1 |  |  | 1 | 3 | 2 | 2 | 2 | 2 |  |  |  |  | **positive** |
|  | Inoculated 2 |  |  | 1 | 1 | 1 |  |  |  |  |  |  |  | **positive** |
| H3N2 | Sentinel 1 |  |  |  | 3 | 2 | 2 | 1 |  |  |  |  |  | **positive** |
|  | Sentinel 2 |  |  | 3 | 3 | 2 |  |  |  |  |  |  |  | **positive** |
|  | Inoculated 1 |  |  | 3 | 3 | 2 | 1 | 1 | 1 | 1 |  |  |  | **positive** |
|  | Inoculated 2 |  |  | 3 | 3 | 3 | 1 |  |  |  |  |  |  | **positive** |

Ferrets were inoculated intranasally with 5 x 10^5^ PFU of indicated viruses in 0.1 mL and the sham group received 100µL PBS. Inoculation was done after isoflurane anaesthesia. Sentinels were added 1 dpi. Clinical scoring: empty cells= normal (i.e. playful and alerted), 1 = mild illness: one clinical sign (inappetence, closed eyes or reduced movement but still alert), 2 = moderate illness: two symptoms (and not playful), 3 = severe illness: nasal discharge, dyspnoea, sneezing, lethargy, shivering and eye closed. Serum samples collected after euthanization 10 dpi were tested after heat inactivation by ELISA. Each sample was tested twice.
